# Characterization of the New Pentafluorosulfanyl-Substituted Chalcone 246TMP-3SF5 as a Potential New Treatment Option Against Hepatocellular Carcinoma

**DOI:** 10.3390/cancers18162640

**Published:** 2026-08-16

**Authors:** Alessandra Viperino, Linda Hammerich, Bernhard Biersack, Supriya Pradhan, Ion Andronache, Isabel Groth, Nicole Edel, Michael Hoepfner, Bianca Nitzsche

**Affiliations:** 1 Institute of Physiology, Charité—University Medical Center Berlin, Corporate Member of Freie Universität Berlin and Humboldt-Universität zu Berlin, Charitéplatz 1, 10117 Berlin, Germany; alessandra.viperino@charite.de (A.V.); isabel.groth@charite.de (I.G.); nicole-edel@t-online.de (N.E.); 2 Department of Hepatology and Gastroenterology, Charité—University Medical Center Berlin, Corporate Member of Freie Universität Berlin and Humboldt-Universität zu Berlin, Augustenburger Platz 1, 13353 Berlin, Germany; linda.hammerich@charite.de; 3 Organic Chemistry Laboratory, University Bayreuth, Universitätsstrasse 30, 95440 Bayreuth, Germany; bernhard.biersack@yahoo.com; 4 Macrogene AI, Vadodara 390009, Gujarat, India; supriyapradhan41660@gmail.com; 5 Advanced Digital Archaeological-Historical Network (ADAHN), Alma Mater Europaea (AMEU), Slovenska Ulica 17, 2000 Maribor, Slovenia; andronacheion@email.su; 6 Fakultät Medizin, MSB Medical School Berlin, Rüdesheimer Straße 50, 14197 Berlin, Germany

**Keywords:** hepatocellular carcinoma, heat shock protein 90, anticancer drug, apoptosis, ferroptosis, cell cycle, migration, chorioallantoic membrane, angiogenesis, fractal analysis, quantitative image analysis

## Abstract

**Background/Objectives**: Advanced-stage hepatocellular carcinoma is characterized by a very poor prognosis; thus, highly effective medication is still needed. Often overexpressed heat shock protein 90 is a promising target due to its pivotal role in carcinogenesis. **Methods**: Antiproliferative effects of 246TMP-3SF5 on HepG2 and HuH-7 cells were assessed by crystal violet staining. Apoptosis was evaluated via subG1 peak, caspase-3 activity and PARP cleavage, and ferroptosis via ROS, glutathione and malondialdehyde levels. Migration was assessed by scratch assay, and in ovo models were used to study angiogenesis and drug effects on microtumors. CAM vascular networks were quantified by semi-automatic segmentation, morphometric and box-counting fractal analysis. Molecular docking and molecular dynamics simulation of heat shock protein 90 were carried out using Autodock Vina and Gromacs respectively. **Results**: Profound dose- and time-dependent antiproliferative effects of 246TMP-3SF5 against HCC cell lines were observed, revealing low micromolecular IC_50_ values and selectivity for carcinoma cells with selectivity indices > 1. A significant increase in the sub-G1 peak, key effector caspase-3 activity, as well as cleavage of PARP strongly suggested apoptosis playing a crucial role in the antiproliferative effects. Additionally, HuH-7 cells revealed an elevation of reactive oxygen species and both cell lines showed significant glutathione depletion concomitant with an increase in malondialdehyde concentration upon treatment. The observed effect could be partially reversed by applying ferrostatin-1, suggesting ferroptosis as an additional relevant mode of action. Changes in the cell cycle as well as impaired tumor cell migration were observed. Upon treatment, angiogenesis was impaired and mass of microtumors was significantly reduced. Quantitative CAM analysis showed that vascularized area fraction increased in controls but decreased under both 17-AAG and 246TMP-3SF5. Likewise global mean vessel width decreased relative to controls, while box-counting dimension was reduced under 246TMP-3SF5. Molecular docking and molecular dynamics simulation analysis predicts 246TMP-3SF5 to be binding in catalytic site of heat shock protein 90. **Conclusions**: 246TMP-3SF5 is a promising novel inhibitor meriting further research as a potential treatment against hepatocellular carcinoma.

## Simple Summary

Hepatocellular carcinoma is the most common form of primary liver cancer, and disease progression often remains unnoticed. However, effective and life-prolonging treatment options for advanced-stage hepatocellular carcinoma are scarce. Tumor cells heavily rely on the heat shock protein family to survive. In particular, heat shock protein 90 is a compelling target in hepatocellular carcinoma, as it promotes tumor growth, invasion and metastasis and correlates with poor prognosis. In this study, a newly synthesized inhibitor derived from the HSP90 inhibitor SU086 was examined in hepatocellular carcinoma cell lines. We found that 246TMP-3SF5 induces cancer cell death by apoptosis and ferroptosis, and in ovo microtumor models confirmed its potent anti-cancer efficacy. Cancer cell migration and formation of new blood vessels were also impaired. 246TMP-3SF5 decreased the vascularized area and reduced vessel width in ovo. These results indicate that 246TMP-3SF5 is a promising novel inhibitor and candidate for further development as treatment against hepatocellular carcinoma.

## 1. Introduction

Hepatocellular carcinoma (HCC) is a highly relevant cancer entity as it represents the most common primary liver cancer and accounts for the third highest cancer death rate worldwide [1,2]. Major risk factors for developing HCC include chronic hepatitis infection, excessive alcohol consumption and metabolic disease leading to non-alcoholic fatty liver disease [1,3,4]. Often HCC remains asymptomatic for a long period of time with symptoms only appearing later on in progressed illness, therefore often leading to a late diagnosis in advanced stages of HCC [5,6]. International therapeutic approaches are based on HCC tumor stages classified by the Barcelona Clinic Liver Cancer (BCLC) staging system depending on tumor size and spread, liver function and physical status of the patient. While early- and intermediate-stage HCC can be treated by a variety of therapeutic options, such as surgical resection, liver transplantation, local ablation or arterial chemoembolization, the most commonly diagnosed advanced-stage HCC is treated with a palliative approach using systemic therapies [4,7,8]. First-line treatment consists of immune checkpoint inhibitors and the antiangiogenic antibody bevacizumab in varying combinations, while multikinase inhibitors, such as sorafenib, are also widely used [1,3,4,8]. However, late diagnosis and underlying chronic liver dysfunction restrict therapy outcomes and resistance of tumors to systemic therapies raises many challenges in the treatment of advanced-stage HCC [9]. Therefore, advanced-stage HCC is still characterized by high lethality and recurrence rate and consequently poor overall prognosis for patients, with median overall survival of only 12–19 months [1,3,8,10,11,12]. Despite the emergence of targeted therapy agents, treatment of advanced-stage HCC remains insufficient, challenged by low response rates and resistance to therapy, resulting in far from satisfactory therapy results [9]. Hence, the development of highly effective and survival-prolonging medication against HCC in advanced stages is highly needed.

Heat shock proteins (HSPs), a family of highly conserved proteins also known as molecular chaperones, are emerging targets for new therapeutic agents. HSPs are ubiquitously expressed and are classified and divided into six major families according to their molecular weight (HSP110, HSP90, HSP70, HSP60, HSP40 and small HSPs). They can be upregulated under conditions of cellular stress (e.g., hypoxia, heat shock, genotoxic agents, nutritional starvation), performing a multitude of functions, such as stabilizing proteins, inhibiting stress-induced protein aggregation and regulating cell signaling and transcription networks [13,14]. Malignant cells seem to be more dependent on the chaperone activity of the heat shock protein family because of their high burden of misfolded and mutated proteins, as well as higher metabolic needs. To facilitate rapid cancer cell proliferation, they exploit these cellular pathways, ensuring cancer cell survival and contributing to tumor progression. Correspondingly, overexpression of HSPs has been observed across multiple cancer entities and linked to therapy resistance, poor prognosis and reduced survival, making them a promising target for cancer therapy [14,15,16,17].

Particularly, heat shock protein 90 (HSP90), with a molecular mass of 90 kDa, is of great interest because of its central role in regulating over 200 client proteins involved in all stages of carcinogenesis. Its client proteins include a wide range of oncoproteins such as kinases (e.g., AKT, RAF), transcription factors (e.g., HIF-1α), as well as hormone receptors, all critical for the growth and survival of cancer cells. Moreover, HSP90 plays a critical role in promoting tumor angiogenesis [18]. HSP90 has been shown to be upregulated in HCC and correlates with a poor prognosis, making it a promising target for new therapeutic approaches, especially in advanced-stage HCC [13,19,20,21,22]. Several HSP90 inhibitors have been developed and tested in various cancer entities, the geldanamycin derivative 17-AAG being one of its prominent examples, inhibiting the function of HSP90 by binding to its ATP-binding pocket, leading to the degradation of various client proteins. Although preclinical studies showed good effectiveness of 17-AAG, its clinical translation was highly challenged by poor solubility and oral bioavailability as well as hepatotoxic side effects [16,23]. Hence, HSP90 inhibitors display a promising target in targeted therapy against HCC but novel inhibitors need to be developed and evaluated.

**Figure 1:**
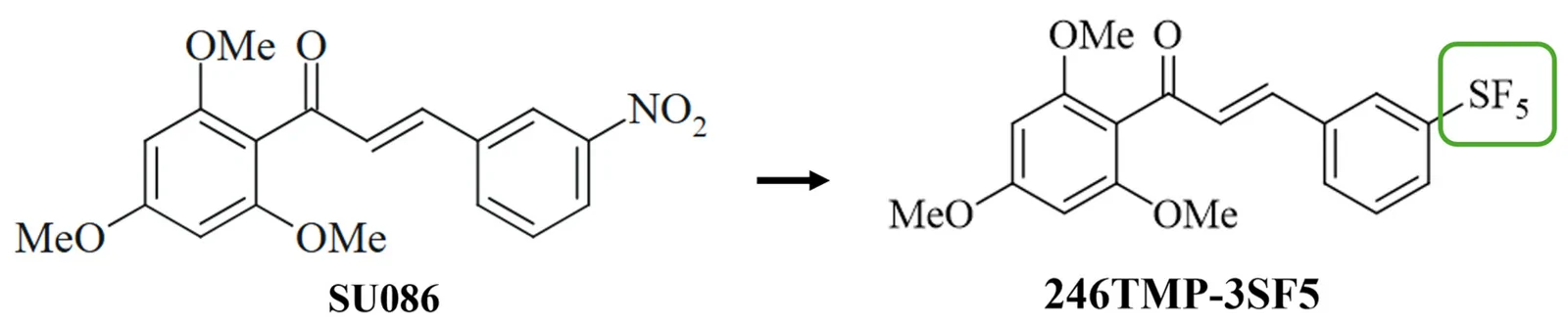
Chemical structure of HSP90 inhibitor SU086 and novel inhibitor 246TMP-3SF5. The figure indicates the chemical modification of the original compound SU086 to obtain the newly synthesized inhibitor. The phenyl–NO_2_ group of SU086 is substituted by a phenyl–SF_5_ group in 246TMP-3SF5 (indicated by the green frame). Both compounds contain a chalcone backbone.

In a previous study, a panel of new inhibitors was investigated [24]. The new compounds were derived from the small molecule SU086, an HSP90 inhibitor shown to be highly effective in prostate cancer [25]. Chemical modifications of the original drug resulted in altered anticancer effects in HCC cell lines. Amongst the panel of newly synthesized compounds, 246TMP-3SF5 proved to be the most effective against HCC cells with the lowest IC_50_ values. 246TMP-3SF5 contains a chalcone backbone (2,4,6-trimethoxyphenyl moiety) with a 3-SF_5_-substituted phenyl group (Figure 1). Chalcones represent a privileged structure in medicinal chemistry, widely used as a promising chemical scaffold for drugs against various disorders, especially various cancers [26]. Both natural and synthetic chalcones have been extensively investigated for anticancer effectiveness and have demonstrated promising results against various cancer types, including colon, breast, lung and prostate cancer [27,28,29]. Mechanisms of anticancer activity of chalcones include apoptosis induction, inhibition of tubulin polymerization, as well as anti-angiogenic and anti-inflammatory effects by targeting multiple cellular molecules, such as MDM2/p53, tubulin, NF-κB, VEGF, EGFR, HIF-1 and many more [28]. Modulating the basic structure of chalcones allows for the synthesis of new compounds with improved anticancer activity [28,29]. The combination of a 2,4,6-trimethoxyphenyl moiety with a 3-SF_5_-substituted phenyl group (246TMP-3SF5) has significant potential as an anticancer drug, as the pentafluorosulfanyl group has been shown to improve compound stability and biological activity [30,31]. In HCC cell lines 246TMP-3SF5 showed pronounced antiproliferative effects with low micromolecular IC_50_ values after 48 h. Long-term assessment of the antiproliferative efficacy also revealed powerful effects on long-term proliferation, with close to no colonies formed after growth for 14 days in both HepG2 and HuH-7 cells. Moreover, nonspecific cytotoxic effects could be excluded as predominant mode of action. Amongst the panel of novel inhibitors evaluated, 246TMP-3SF5 proved to be the most active compound and was therefore chosen as most promising candidate for further research [24].

In this work, we further investigated the underlying mechanism of action of 246TMP-3SF5 in HCC cell lines HepG2 and HuH-7. Additionally, experiments were performed to examine the effects of the new drug on cell cycle regulation and crucial hallmarks of cancer cells, such as cell migration and induction of angiogenesis. Furthermore, we evaluated the antineoplastic efficacy of the novel inhibitor on HCC microtumors in ovo to assess its effectiveness as a potential novel drug in the treatment of HCC.

## 2. Materials and Methods

### 2.1. Compounds

The synthesis of the pentafluorosulfanyl-substituted chalcone 246TMP-3SF5 was published before [24]. The HSP90 inhibitor 17-AAG was purchased from AbMole BioScience (Houston, TX, USA, Cat. No. M2320) and the multikinase inhibitor sorafenib was purchased from TargetMol (Boston, MA, USA, Cat. No. T0093L). Inducer of ferroptotic cell death, erastin, was purchased from TargetMol (Boston, MA, USA, Cat. No. 571203-78-6) and the ferroptosis inhibitor ferrostatin-1 was purchased from Bio-Techne GmbH (Wiesbaden-Nordenstadt, Germany, Cat. No. 5180). 20 mM stock solutions of the compounds were prepared using dimethyl sulfoxide (DMSO, Sigma-Aldrich, Darmstadt, Germany) and stored at −20 °C. Working solutions (1 mM) were freshly prepared for the experiments by diluting the stock solution with cell culture medium and stored at 4 °C for up to two weeks.

### 2.2. Cell Culture

Human hepatocellular carcinoma cell line HuH-7 (JCRB number: JCRB0403) and human hepatoma cell line HepG2 (ATCC number: HB-8065) were cultured as previously described [24]. In brief, cells were cultured in RPMI 1640 medium supplemented with 10% fetal bovine serum, 100 U/mL penicillin, 100 µg/mL streptomycin and 2 mM L-glutamine. The non-malignant endothelial cell line EA.hy 926 (ATCC number: CRL-2922) was cultured in DMEM medium supplemented with 10% fetal bovine serum, 100 U/mL penicillin and 100 µg/mL streptomycin. All cell lines were cultured at 37 °C, 5% CO_2_, in a humidified atmosphere in an incubator.

### 2.3. Antiproliferative Assessment by Crystal Violet Assay

Treatment-induced antiproliferative effects on various cell lines were measured as a decrease in cell count assessed by crystal violet (hexamethyl-*para*-rosaniline chloride from Sigma-Aldrich, Darmstadt, Germany) staining as previously described [24,31,32]. In brief, HepG2, HuH-7 and EA.hy 926 cells seeded in 96-well plates were treated for 24, 48 and 72 h with increasing concentrations of SU086, 246TMP-3SF5, 17-AAG or sorafenib; after fixation with 1% glutaraldehyde, staining of the cells was performed with 0.1% crystal violet for 30 min. Plates were rinsed with running water to remove unbound dye and cells were lysed by incubation with 0.2% Triton X-100 overnight. Absorbance was measured at 570 nm (microplate reader from Dynex Technologies, Denkendorf, Germany; GloMax^®^ Discover Microplate Reader Promega, Madison, WI, USA). Non-linear regression analysis was performed and dose-response curves generated. Calculated IC_50_ values are shown. Selectivity Indexes were calculated as SI_a_ = IC_50_ EA.hy 926/IC_50_ HepG2 cells; SI_b_ = IC_50_ EA.hy 926/IC_50_ HuH-7 cells. A minimum of *n* ≥ 3 independent experiments were performed in triplicate or more for each cell line.

### 2.4. Caspase-3 Activity Assessment

To assess drug-induced apoptosis, a caspase-3 assay was performed using Ac-DEVD-AMC (Cayman Chemical, Ann Arbor, MI, USA, Cat. No. 14986), a substrate that exhibits a fluorogenic signal upon cleavage by active caspase-3. The assay was performed as previously described [31]. Briefly, cells were seeded at a density of 1 × 10^5^ cells/well in 12-well plates and incubated overnight. Afterwards, the cells were treated with 1.0 µM, 5.0 µM, or 10.0 µM of 246TMP-3SF5 or 17-AAG for 24 h. Cells were washed and frozen, followed by lysis with RIPA buffer for 30 min on ice. Using the PierceTM BCA Protein Assay Kit (Thermo Fisher Scientific, Waltham, MA, USA, Cat. No. 23227), the protein content of the samples was quantified. To detect the activity of caspase-3, the samples were incubated at 37 °C for 1 h with Ac-DEVD-AMC. Substrate cleavage was measured fluorometrically using the GloMax^®^ Discover Microplate Reader (Promega, Madison, WI, USA). The measured fluorescent signal was then normalized to the previously calculated protein levels in each sample. *n* = 3–4 independent experiments were performed in duplicate, and data are given as folds of the control ± SEM.

### 2.5. Western Blot Analysis

Protein expression was analyzed by Western blotting, as reported previously [24]. In brief, HepG2 and HuH-7 cells were treated with increasing concentrations of 246TMP-3SF5 or 17-AAG for 24 h. And then the cells lysed in RIPA buffer mixed with cOmplete™ Mini Protease Inhibitor (Roche, Mannheim, Germany). Total protein in the samples was quantified using the Pierce™ BCA Protein Assay Kit and 20 µg of protein was separated using SDS–PAGE and blotted onto PVDF membranes. After blocking with TBS containing 0.1% Tween-20 and 5% non-fat dry milk, blots were incubated with primary antibodies against PARP (poly-ADP-ribose-polymerase) (Cell Signaling, Boston, MA, USA) and β-actin (Sigma-Aldrich, Germany). HRP-conjugated goat anti-mouse IgG or anti-rabbit IgG (both from LI-COR Biotechnology, Bad Homburg, Germany) were used and chemiluminescent bands were developed using Clarity^TM^ Western ECL Substrate (Bio-Rad, Feldkirchen, Germany) and imaged with ChemiDoc MP System (Bio-Rad Laboratories, Feldkirchen, Germany). *n* = 3 independent experiments were performed.

### 2.6. Cell Cycle Analysis by Flow Cytometry

To determine changes in the cell cycle due to the HSP90 inhibitors, cell cycle analysis was performed using the DNA-staining dye propidium iodide (PI) and flow cytometry. HepG2 and HuH-7 cells were seeded at 5 × 10^3^ cells/well in 6-well plates and incubated overnight and then treated with 246TMP-3SF5 or 17-AAG at the indicated concentrations for 24 h. Cells were washed with PBS and harvested, fixed and permeabilized with 70% ethanol overnight at −20 °C. Thereafter, cells were washed three times with PBS and incubated with RNase A (0.4 mg/mL) for 30 min at 37 °C. After staining the cells with PI (50 µg/mL) (Invitrogen, Eugene, OR, USA) in the dark, flow cytometry was conducted using the FACSCanto II (BD Biosciences, Heidelberg, Germany). A total of 50,000 single cells were measured and analyzed using FlowJo software (version 10.10.0; LLC, Ashland, OR, USA). *n* = 4 independent experiments were performed; representative histograms and data as means ± SEM are shown.

### 2.7. Reactive Oxygen Species Detection

To assess the formation of cytosolic reactive oxygen species (ROS), the cell membrane-permeable dye CellROX^®^ Orange (Thermo Fisher Scientific, Waltham, MA, USA, Cat. No. C10443) was used as previously described [31]. The dye exhibits a strong red fluorescent signal upon oxidation at excitation/emission levels of 545 nm/565 nm. In brief, cells were seeded (1 × 10^5^ cells/well for HepG2, 2 × 10^5^ cells/well for HuH-7) in 12-well plates and allowed to attach overnight. The cells were then treated with 246TMP-3SF5, 17-AAG or erastin at the indicated concentrations. CellROX^®^ Orange (1 μM) was applied together with the drugs. Following an incubation time of 24 h, fluorescent images were taken using a ZOE™ Fluorescent Cell Imager (Bio-Rad Laboratories, Munich, Germany). Untreated cells served as the negative control, while untreated cells incubated with 1.6 mM H_2_O_2_ for 30 min prior to imaging served as the positive control. *n* ≥ 3 independent experiments were performed, and representative images are shown.

### 2.8. Measurement of Intracellular Glutathione Levels

To determine changes in intracellular glutathione levels, the GSH-Glo™ Glutathione Assay was performed (Promega, Madison, WI, USA, Cat. No. V6911) according to the manufacturer’s instructions. In brief, HCC cells were seeded at 3 × 10^6^ cells/well and allowed to adhere overnight before being treated with 246TMP-3SF5, 17-AAG or erastin at the indicated concentrations for 24 h. Intracellular glutathione drives the formation of luciferin, which then produces light directly proportional to the amount of glutathione present. Luminescence was measured using the GloMax^®^ Discover Microplate Reader (Promega, Madison, WI, USA). *n* = 3–4 independent experiments were performed in duplicate, and data are given as the mean percentage compared to the control (set to 100%) ± SEM.

### 2.9. Malondialdehyde Analysis

To assess lipid peroxidation the Malondialdehyde (MDA) Colorimetric Assay Kit was purchased from Elabscience (Houston, TX, USA, Cat. No. E-BC-K028-M). The assay was used according to the manufacturer’s instructions. In brief, cells were treated with 246TMP-3SF5, 17-AAG, erastin and ferrostatin-1 at the indicated concentrations for 24 h. Afterwards cells were harvested, homogenized and incubated with thiobarbituric acid at 100 °C for 40 min. The thiobarbituric acid reacts with MDA to produce a pinkish coloring, which can be measured at absorption of 532 nm. Protein levels of samples were measured using Pierce™ BCA Protein Assay Kit (Thermo Fisher Scientific, Waltham, MA, USA, Cat. No. 23227). Measured absorbance was normalized to the protein level of the samples, and the MDA concentrations were calculated using the provided standard solution. Erastin (10 µM) served as a positive control. *n* = 3–5 independent experiments were performed, and data are shown as means ± SEM compared to the control (set to 100%).

### 2.10. Scratch Assay

To investigate anti-migratory effects of 246TMP-3SF5, HepG2 and HuH-7 cells were seeded in 6-well plates and allowed to grow until sub-confluency. The cell monolayer was scratched vertically with a 10 µL pipette tip and detached floating cells were removed by gently washing with PBS. Cells were then incubated for up to 24 h with rising concentrations of 246TMP-3SF5 (1.0–10.0 µM) or 10.0 µM 17-AAG, respectively. Untreated cells served as the control. Pictures at t = 0 h and t = 24 h were taken using the EVOS M5000 microscope (Thermo Fisher Scientific, Waltham, MA, USA). The scratch area was quantified using the wound healing size tool plug-in for ImageJ (version 1.54p, National Institutes of Health, USA). Cell migration was calculated using the following formula

$$\begin{array}{c} Cell\;Migration\;Rate\;\left[\%\right]\\ =\left(\frac{initial\;scratch\;area\;\left[\%\right]-scratch\;area\;after\;24\;h\;\left[\%\right]}{initial\;scratch\;area\;\left[\%\right]}\right)\times 100\%. \end{array}$$

Quantified cell migration rate in percentage as means ± SEM and representative images of *n* = 3 independent experiments are shown.

### 2.11. In Ovo Assessment of Antiangiogenic and Antineoplastic Effects

To examine anti-angiogenic and anti-neoplastic effects of 246TMP-3SF5 in a systemic setting, in ovo experiments utilizing the chorioallantoic membrane (CAM) of fertilized chicken eggs were conducted as previously described [31,32,33]. Fertilized chicken eggs (*Gallus gallus*) were incubated at 37.8 °C and >58% humidity. On day 3–4 of embryonic development, a small hole was pierced into the poles of the egg and 1–2 mL of albumin was removed, causing the CAM to detach from the eggshell and lower itself down into the allantoic cavity.

To assess anti-angiogenic effects changes in the vascularization of the chorioallantoic membrane (CAM) upon treatment were analyzed. On day 11 of embryonic development, the eggshell was carefully opened to place a sterilized silicone ring (Ø 5 mm) on the region of interest on the CAM. Afterwards, 20 µL of 0.9% NaCl, which served as a control, or compound diluted in 0.9% NaCl was added into the ring at the indicated concentrations. Changes in the angiogenesis and vascularization of the CAM were documented by taking pictures using a light microscope equipped with a Kappa digital camera system (Distelkamp-Electronic, Kaiserslautern, Germany) after 24 and 48 h. Representative images are shown. At least *n* = 5 eggs for each condition were assessed.

To assess anti-neoplastic effects of the novel compound, 3 × 10^6^ HuH-7 tumor cells suspended in 20 µL Matrigel were implanted on the chorioallantoic membrane of the fertilized chicken eggs on day 8 of embryonic development using a silicone ring of 5 mm in diameter. The tumor-bearing eggs were incubated for 24 h, allowing the tumors to attach and connect to the microvessels of the CAM, following a topical application of 20 µL of 246TMP-3SF5 or 17-AAG for 72 h at the indicated concentrations. Viability of the chicken embryo and tumor growth were controlled daily. Pictures of the tumors were taken using a microscope (Kappa digital camera system, Distelkamp-Electronic, Kaiserslautern, Germany). Thereafter, the tumors were carefully cut out and weighed. Representative images are shown and tumor weight after treatment is quantified as means ± SEM. A minimum of *n* = 5 eggs were analyzed for each condition.

### 2.12. Morphometric and Fractal Analysis of Chorioallantoic Membrane Images

CAM images were analyzed at their native 640 × 480-pixel resolution using a semi-automatic segmentation workflow with expert quality control. The region of interest (ROI) was defined as the interior of the silicone ring using ellipse fitting with audited frame-specific corrections. Candidate vessels were identified from local green-channel darkening, red-excess features and multiscale Hessian ridge responses, followed by connected propagation from high-confidence vascular seeds and spatial tracking of pale linear branches within each image; no temporal interpolation was applied. Final masks were strictly binary and were analyzed without resizing, gap filling or pruning. Skeletonization preserved the topology of the vascular mask in all 51 images (identical component and cycle counts), and 18,292 individual branches were measured; excluding terminal spurs ≤ 3 px altered total skeleton length by 0.06–1.15% per image.

Vascularized area fraction (VAF, % of ROI) was calculated as 100× the number of vascular pixels divided by the total number of pixels within the ROI and served as the primary quantitative endpoint. Vessel caliber was quantified as vascular area divided by skeleton graph length (px), and fractal organization via box-counting dimension (Db, 1–32 px, with regression quality audited per image); both served as complementary endpoints. As skeletons were not pruned, branch counts are sensitive to contour roughness and caliber and were not used as a measure of vascular extent. All lengths/widths are in pixels. Original data and results of CAM analysis can be accessed via the Google Drive depository.

In total, 51 masks from 17 biological samples (control *n* = 5, 17-AAG *n* = 5, 246TMP-3SF5 *n* = 7) were evaluated at 0, 24 and 48 h, with the biological sample as the longitudinal unit. Control egg04 was flagged by image quality control due to exposure/white-balance inconsistencies and was retained in the primary summary, with an additional sensitivity analysis. Analyses used Python 3.12.13 (NumPy 2.3.5, SciPy 1.17.0, Pillow 12.2.0, Matplotlib 3.10.8).

### 2.13. Molecular Docking

The docking studies of 17-AAG and 246TMP-3SF5 bound to the ATP binding site of HSP90 (PDB ID 1YET) were performed to determine their binding mode [34]. Protein and ligand preparation and visualization were done using UCSF Chimera version 1.16 [35]. For docking AutoDock Vina version 1.1.2 was used [36]. The crystal structure from PDB ID 1YET with 1.90Å resolution was extracted and opened in UCSF Chimera. All the water molecules were removed and the protein and ligand molecules were separately prepared. Preparation of the protein was carried out by adding Hydrogens, missing side chains, and Gasteiger charges [37]. All the ligand molecules were prepared, converted to PDBQT files and docked using AutoDock Vina.

### 2.14. Molecular Dynamics Simulation

We performed 100 ns MD simulation of 246TMP3SF5 docked into ATP-binding site of the HSP90 protein using Gromacs [38]. The system was parameterized using the Amber forcefield and solvated in 10Å cubic box with the TIP3 model. Counter ions added to neutralize the system. Energy minimization was done using the steepest descent method. Thermostats were kept at 300K with NVT and 1 atm pressure for the NPT ensemble; each run was done for 100 ps and the production run was 100 ns. The simulation was forwarded to the production run after the temperature and pressure equilibration was attained.

### 2.15. Statistical Analysis

Statistical analyses were performed using GraphPad Prism (v10.6.1, La Jolla, CA, USA). Data sets were compared using ordinary one-way ANOVA followed by Dunnett’s or Tukey’s multiple comparisons tests. Statistical significance was assumed at * *p*  <  0.05, ** *p*  <  0.01, *** *p*  <  0.001 and **** *p*  <  0.0001. Data are presented as mean ± SEM unless otherwise stated.

## 3. Results

### 3.1. Antiproliferative Effects of 246TMP-3SF5 Against Human Liver Cancer Cells

Previously amongst a panel of different inhibitors tested, the novel compound 246TMP-3SF5 exerted selective antiproliferative effects on liver cancer cells with low micromolecular IC_50_ values of 1.3 µM (±0.2 µM) in HepG2 and 2.2 µM (±0.3 µM) in HuH-7 cells after 48 h of treatment. In comparison, the established HSP90 inhibitor 17-AAG showed similar IC_50_ values of 1.6 µM (±0.5 µM) and 0.4 µM (±0.4 µM) for HepG2 and HuH-7 cells respectively. However, sorafenib, currently used as a chemotherapeutic agent in first-line treatment of advanced-stage HCC, reached IC_50_ values of 4.4 µM (±1.0 µM) in HepG2 cells and 5.4 µM (±0.6 µM) in HuH-7 cells [24].

**Figure 2:**
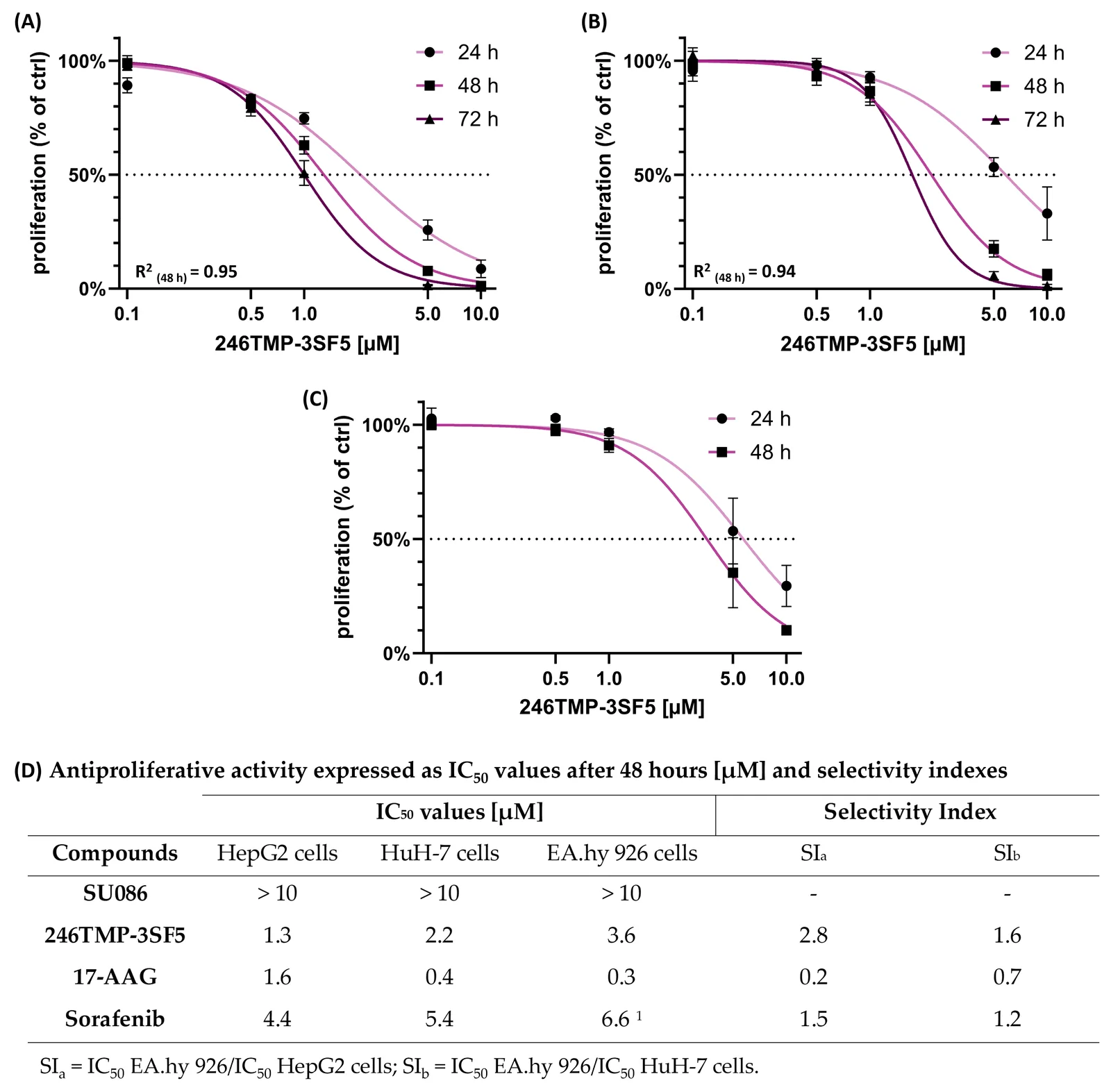
Antiproliferative effects of 246TMP-3SF5 against HCC cell lines. Crystal violet assay demonstrated pronounced time- and dose-dependent antiproliferative effects against HCC cell lines. Dose-dependent inhibition of cell proliferation after 24, 48 and 72 h of treatment for HepG2 (**A**) and HuH-7 (**B**) cells can be observed. Non-malignant cell line EA.hy 926 was assessed as well (**C**). Data is shown as means ± SEM and curves were generated through non-linear regression analysis. IC_50_ values after 48 h of treatment and selectivity indices are shown in the table (**D**). At least *n* ≥ 3 independent experiments were performed in triplicate or more. ^1^ IC_50_ value for sorafenib in EA.hy 926 cells was taken from [39].

The antiproliferative effect of 246TMP-3SF5 was time- and dose-dependent in both HCC cell lines (Figure 2A,B). It is noteworthy that increased treatment time enhanced the antiproliferative effects; however, the differences could only be observed at concentrations starting from 1.0 µM and are modest at 5.0 and 10.0 µM after 48 and 72 h. The dose-dependently increased antiproliferative effectiveness is more pronounced, following a sigmoid curve. In HepG2 cells the effect of the novel inhibitor is more prominent: 1.0 µM 246TMP-3SF5 inhibits proliferation down to 50% compared to control after 72 h. Interestingly, the antiproliferative efficacy reached a peak at 5.0 µM 246TMP-3SF5 at 72 h. After 72 h HCC cell proliferation upon treatment amounted to only 1.5% (±0.3%) of HepG2 cells and 5.9% (±1.8%) of HuH-7 cells. While a twofold increase in concentration to 10.0 µM 246TMP-3SF5 did increase the antiproliferative effect further to almost no viable cells left, the effect at 5.0 µM is already profound.

To investigate the effect of 246TMP-3SF5 on non-malignant cells, a crystal violet assay was performed with endothelial EA.hy 926 cells. Antiproliferative assessment revealed that 246TMP-3SF5 impairs cell proliferation with an IC_50_ value of 3.6 µM (Figure 2C,D). To compare the effect of the novel inhibitor between HCC and non-malignant cells, a selectivity index (SI) was calculated. SI_a_ for HepG2 cells amounted to 2.8 and SI_b_ to 1.6 in HuH-7 cells (Figure 2D). For comparison, antiproliferative effects in all cell lines and SI were also evaluated for SU086, 17-AAG and sorafenib (Supplementary Figure S1 and Figure 2D). While SU086 did not suppress cell proliferation in either HCC and endothelial cells, sorafenib showed SI indexes > 1; however, slightly lower than those of 246TMP-3SF5. Noticeably, SI indexes for 17-AAG were below 1, with SI_a_ of 0.2 in HepG2 and SI_b_ of 0.7 in HuH-7 cells (Figure 2D).

In line with the antiproliferative effects observed, 246TMP-3SF5 was chosen as a promising novel inhibitor for further investigations because of its low micromolecular IC_50_ values in HCC cell lines and preferable SI indices. Regarding the investigation of the mode of action, concentrations of 1.0, 5.0 and 10.0 µM 246TMP-3SF5 were used for the following experiments.

### 3.2. Apoptotic Effects of 246TMP-3SF5 in HCC Cell Lines

To investigate the mode of action of the novel HSP inhibitor 246TMP-3SF5 in HCC cell lines, apoptotic cell death was evaluated as a possible mechanism of action explaining the antiproliferative effects of the novel inhibitor. Investigation of apoptosis was conducted via assessment of the sub-G1 peak via flow cytometry, caspase-3 activity assay, as well as western blot assessment of poly-(ADP-ribose)-polymerase (PARP). Dose-dependent effects of 246TMP-3SF5 were evaluated throughout the experiments. 17-AAG (tanespimycin) was used as a reference HSP90 inhibitor for comparison.

**Figure 3:**
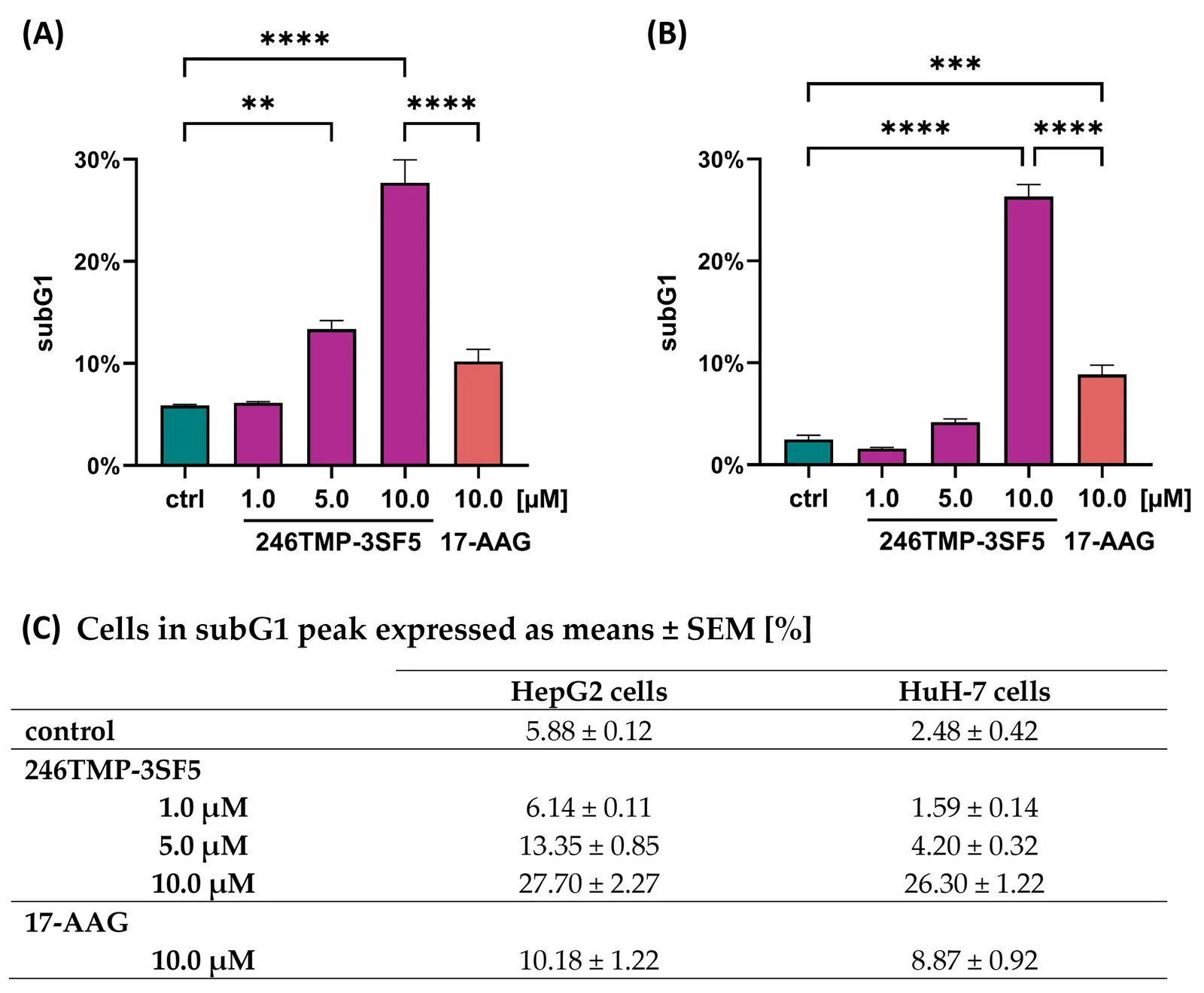

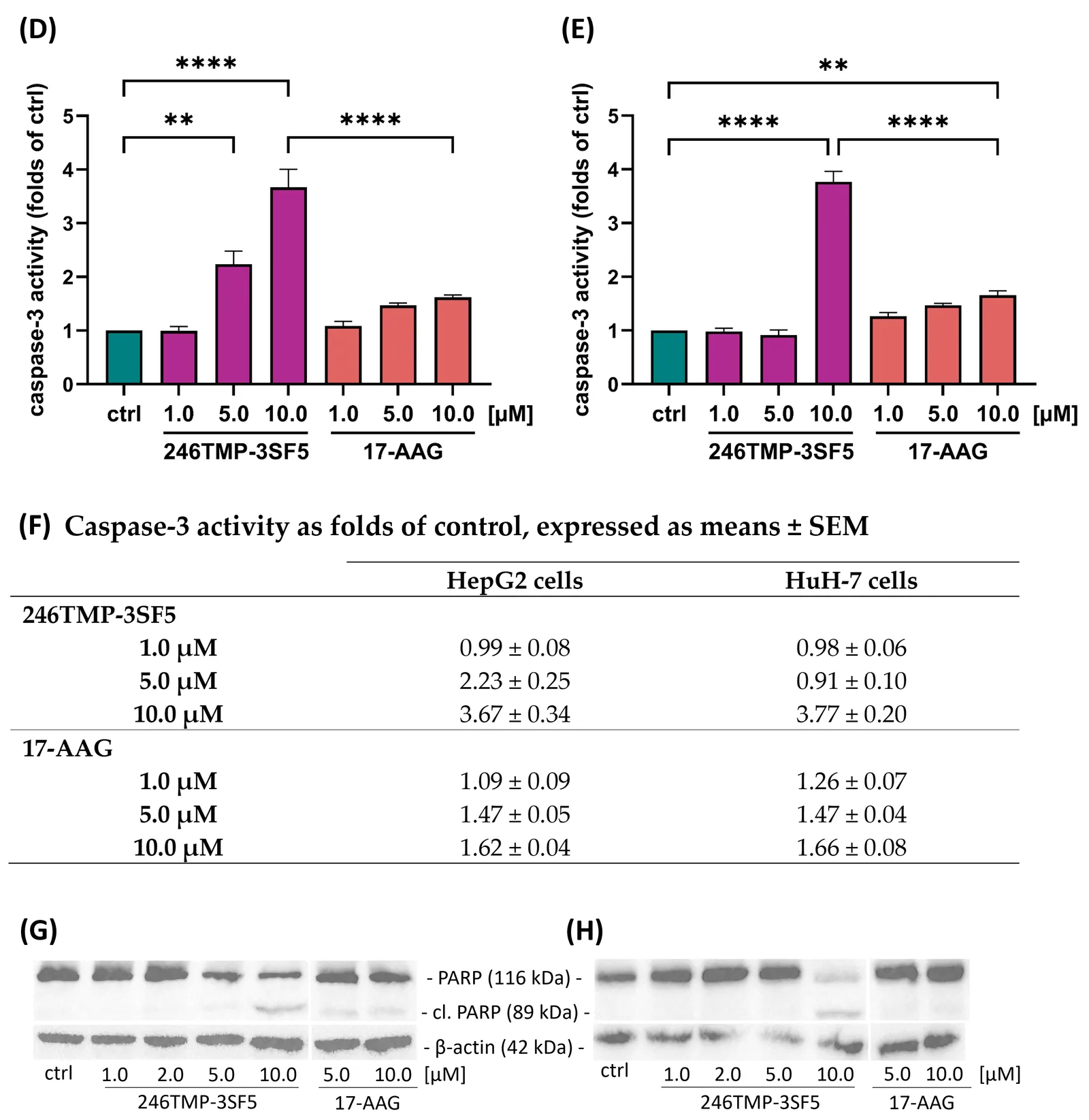
246TMP-3SF5 induces apoptosis in HCC cell lines. SubG1 peak increased significantly following 246TMP-3SF5 treatment for 24 h in HepG2 (**A**) and HuH-7 cells (**B**) in a dose-dependent manner, assessed through flow cytometry. *n* = 4 independent experiments were conducted. Data is shown in table as means ± SEM (**C**). Caspase-3 activity is significantly elevated upon treatment with inhibitor 246TMP-3SF5 after 24 h in HepG2 (**D**) and HuH-7 cells (**E**). *n* ≥ 3 independent experiments were performed. Table shows data expressed as means ± SEM, as folds of the control set to 1 (**F**). Statistical significance was assessed by ordinary one-way ANOVA followed by Tukey’s multiple comparisons test. Significance levels are shown as follows: ** *p* ≤ 0.01, *** *p* ≤ 0.001, **** *p* ≤ 0.0001. Dose-dependent increase in cleaved poly-(ADP-ribose)-polymerase (cl. PARP) following treatment with 246TMP-3SF5 at indicated concentrations for 24 h in HepG2 (**G**) and HuH-7 cells (**H**) was observed. *n* = 3 independent Western blots were generated; representative images are shown. The original blot images can be found in Supplementary File S1.

246TMP-3SF5 exhibited a significant dose-dependent increase in cells in the sub-G1 peak in both HCC cell lines after treatment for 24 h; the effect was significantly more pronounced than that for 17-AAG at equal concentration (Figure 3A–C). SubG1 peak reached a mean of up to 27.7% (±2.3%) in HepG2 and 26.3% (±1.2%) in HuH-7 cells upon treatment with 10.0 µM 246TMP-3SF5 (Figure 3C). These findings suggest that 246TMP-3SF5 increased the number of dead cells and DNA fragments upon treatment. Therefore, apoptosis was further investigated as potential mode of action by analyzing key enzymes of the apoptosis cascade. We found caspase-3, a key effector caspase during apoptosis, to be significantly elevated after treatment with the novel inhibitor 246TMP-3SF5 for 24 h (Figure 3D–F). The effect showed a dose-dependent pattern for HepG2 cells, with both HCC cell lines reaching a peak of caspase-3 activity of more than threefold compared to untreated controls at 10.0 µM 246TMP-3SF5. While at high concentrations 17-AAG also slightly increased caspase-3 activity by up to 1.6-fold (±0.04) in HepG2 and 1.7-fold (±0.1) in HuH-7 cells compared to control, the effect following treatment with 246TMP-3SF5 was significantly more pronounced. In comparison, the novel inhibitor increased caspase-3 activity by up to 3.7-fold (±0.3) in HepG2 and 3.8-fold (±0.2) in HuH-7 cells at equal concentration of 10.0 µM (Figure 3F). Western blot analysis revealed a decrease in full-length PARP and cleavage of PARP following treatment with 10.0 µM 246TMP-3SF5 for 24 h in both HCC cell lines. 17-AAG also induced the cleavage of PARP in HepG2 cells, said effect only faintly noticeable in HuH-7 cells (Figure 3G,H).

### 3.3. Assessment of Cellular Stress and Ferroptotic Cell Death in HCC Cell Lines Treated with 246TMP-3SF5

To further decipher the underlying molecular mode of action of the antiproliferative effects of 246TMP-3SF5, ferroptotic cell death was assessed. Ferroptosis is a form of regulated cell death characterized by an iron-dependent accumulation of lipid peroxides and subsequent plasma membrane damage of cells, driven by an accumulation of reactive oxygen species (ROS) and dysregulation of antioxidative systems, such as the glutathione–glutathione peroxidase 4 pathway [40,41,42]. The ferroptosis inducer erastin was used as a positive control.

**Figure 4:**
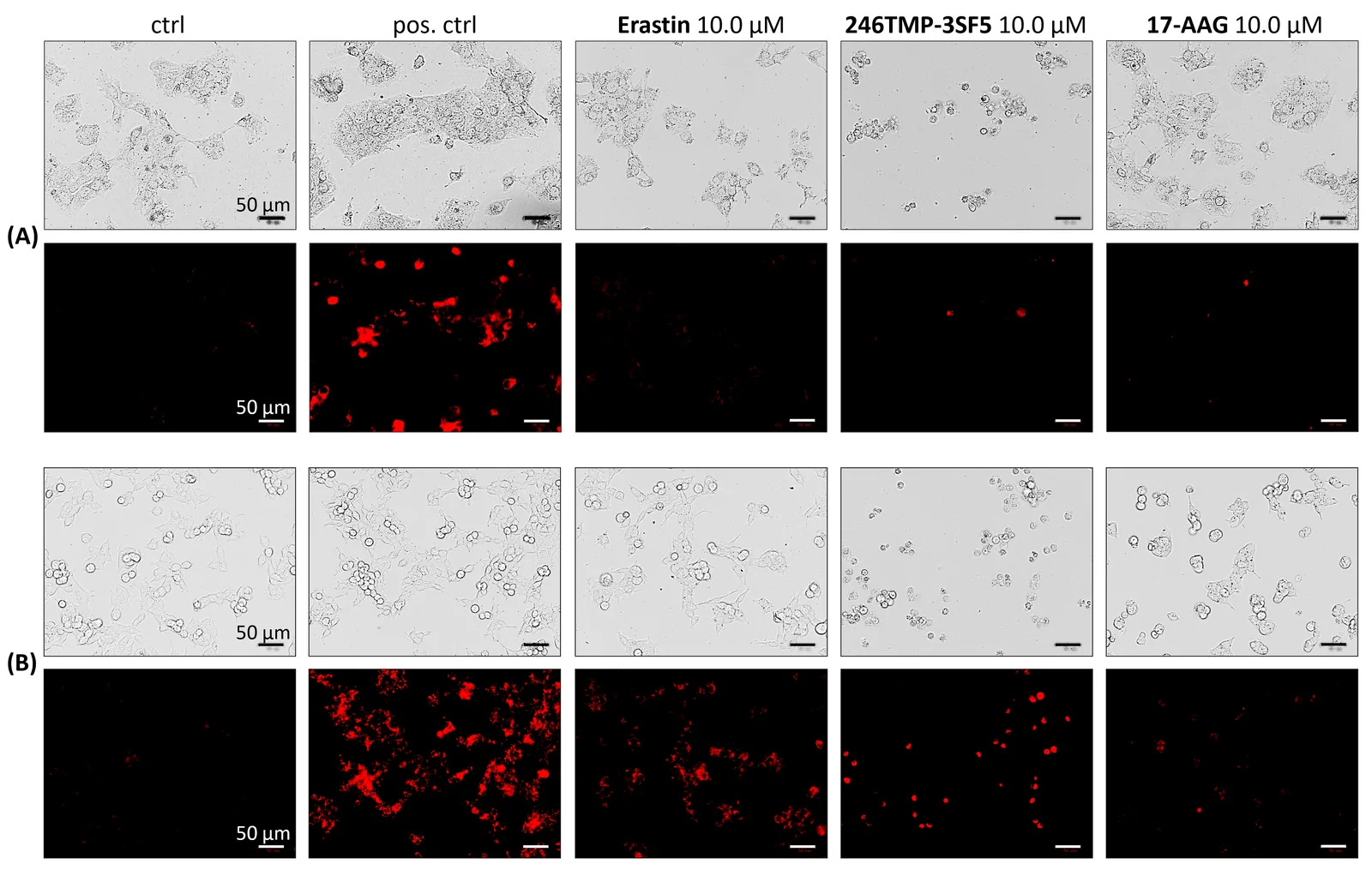
Intracellular reactive oxygen species increase following 246TMP-3SF5 treatment. Reactive oxygen species (ROS) levels upon treatment with 246TMP-3SF5 for 24 h were assessed in HepG2 (**A**) and HuH-7 cells (**B**) using a red fluorescent dye. Untreated cells incubated with 1.6 mM H_2_O_2_ for 30 min served as positive control. Images show brightfield and corresponding red fluorescent images. *n* ≥ 3 independent experiments were conducted and representative images are shown.

Firstly, ROS induction by 246-TMP-3SF5 in HCC cells was investigated (Figure 4). Upon treatment with 246TMP-3SF5 for 24 h, an increase in fluorescent red-labeled cells due to a rise in intracellular ROS levels compared to the control became evident. ROS induction by 246TMP-3SF5 was more pronounced in HuH-7 cells than in HepG2 cells (Figure 4B). Erastin showed a similar effect to 246TMP-3SF5; its impact on ROS accumulation was clearly noticeable in HuH-7 cells as well. In contrast, 17-AAG induced a less intense ROS accumulation in HuH-7 cells. These findings indicate that 246TMP-3SF5 increased intracellular ROS levels predominantly in HuH-7 cells, similar to the ferroptosis inducer erastin.

Glutathione (GSH) levels and malondialdehyde (MDA) concentrations of HCC cells treated with the novel inhibitor were analyzed to further evaluate ferroptosis as a relevant mode of action. GSH is a cysteine-containing tripeptide playing an essential role as an electron donor in antioxidative systems of the cell, suppressing ferroptosis. Low GSH levels cause an imbalance in antioxidative systems, impairing their function and leading to ferroptosis [41,42,43]. Erastin induces ferroptosis by blocking the main channel responsible for cellular cysteine import, which serves as a building block for GSH [41]. Moreover, reactive aldehydes formed through lipid peroxidation, such as MDA, are crucial indicators of ferroptosis [44].

**Figure 5:**
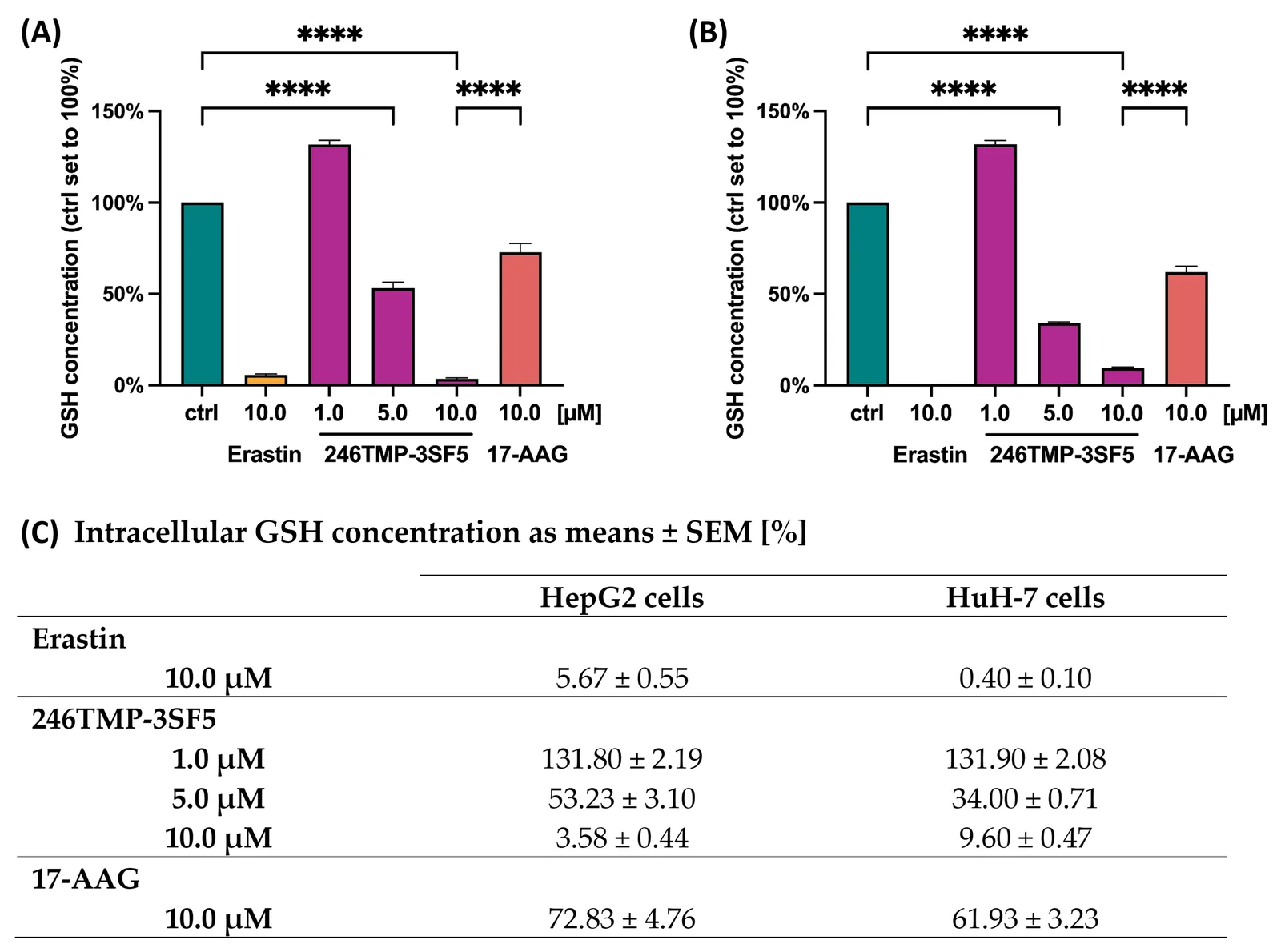

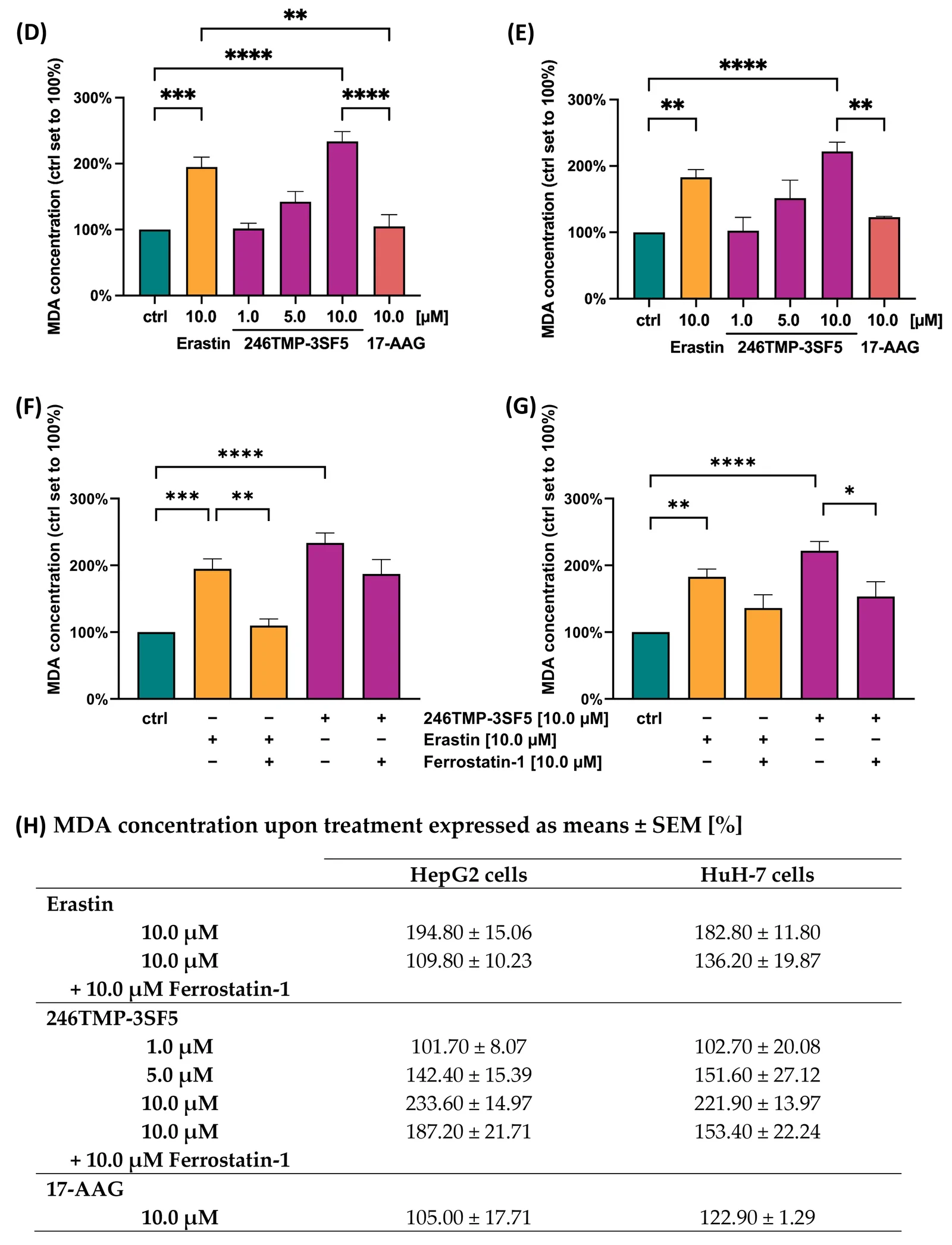
246TMP-3SF5 induces ferroptotic cell death in HCC cells. Intracellular GSH levels significantly decreased after treatment with 246TMP-3SF5, while MDA concentrations rose following treatment in HepG2 (**A**,**D**) and HuH-7 cells (**B**,**E**) in a dose-dependent manner. The effect is partially reversible by ferrostatin-1 in both HepG2 (**F**) and HuH-7 cells (**G**). GSH and MDA levels were quantified upon treatment with 246TMP-3SF5, the ferroptosis inducer erastin, HSP90 reference inhibitor 17-AAG and ferroptosis inhibitor ferrostatin-1 and the tables show a summary of the data as means ± SEM compared to the control set to 100% (**C**,**H**). *n* = 3–4 independent experiments were performed in duplicate for GSH analysis and *n* ≥ 3 independent experiments were performed for MDA assessment. Data are shown as means ± SEM compared to the control set to 100%. Ordinary one-way ANOVA followed by Tukey’s multiple comparisons test was performed and significance levels are displayed as * *p* ≤ 0.05, ** *p* ≤ 0.01, *** *p* ≤ 0.001, **** *p* ≤ 0.0001.

Assessment of intracellular GSH levels revealed a significant dose-dependent GSH depletion in both HCC cell lines following treatment with 246TMP-3SF5 (Figure 5A–C). Interestingly, the results showed that 1.0 µM 246TMP-3SF5 increased GSH levels compared to the control. Strikingly, 10.0 µM 246TMP-3SF5 significantly decreased intracellular GSH levels to 3.6% (±0.4%) in HepG2 and 9.6% (±0.5%) in HuH-7 cells compared to the control. Similar effects were observed for 10.0 µM erastin, with GSH levels of 5.7% (±0.6%) for HepG2 and 0.4 (±0.1%) for HuH-7 cells. On the other hand, 10.0 µM 17-AAG also caused a reduction in GSH levels; however, the effect was not as pronounced, with GSH levels of 72.8% (±4.8%) and 61.9% (±3.2%) in HepG2 and HuH-7 cells respectively (Figure 5C). Comparing both inhibitors, 246TMP-3SF5 significantly exceeded ferroptosis-associated GSH level reduction in HCC cells (Figure 5A,B). The results indicate that oxidative stress and imbalance of antioxidative systems might also play a role in the mechanisms behind the antiproliferative effect of 246TMP-3SF5 in HCC cell lines.

Furthermore, changes in MDA levels were analyzed. Upon treatment with 246TMP-3SF5 a significant dose-dependent increase in MDA levels was observed in both cell lines, suggesting that ferroptosis is a relevant form of cell death occurring due to 246TMP-3SF5 treatment (Figure 5D,E,H). MDA levels reached a mean of 233.6% (±15.0%) in HepG2 and 221.9% (±14.0%) in HuH-7 cells at 10.0 µM 246TMP-3SF5 compared to untreated controls. Notably, the increase in MDA due to 17-AAG turned out to be minor at 105.0% (±17.7%) in HepG2 and 122.9% (±1.3%) in HuH-7 cells, in line with the previously assessed GSH depletion not being as pronounced (Figure 5H). It is noteworthy that while both erastin and the novel inhibitor led to a significant increase in MDA levels in both HCC cell lines at 10.0 µM, the effect is slightly more pronounced for 246TMP-3SF5. Thereafter reversibility of the observed effect was assessed by co-incubation with the ferroptosis inhibitor ferrostatin-1. Increase in MDA levels was partially reversible for erastin and 246TMP-3SF5 in both HCC cell lines upon co-incubation with ferrostatin-1 (Figure 5F–H). The effect was significant in HuH-7 cells for the novel inhibitor (Figure 5G).

### 3.4. Evaluation of Cell Cycle Regulation upon 246TMP-3SF5 Treatment in HCC Cell Lines

**Figure 6:**
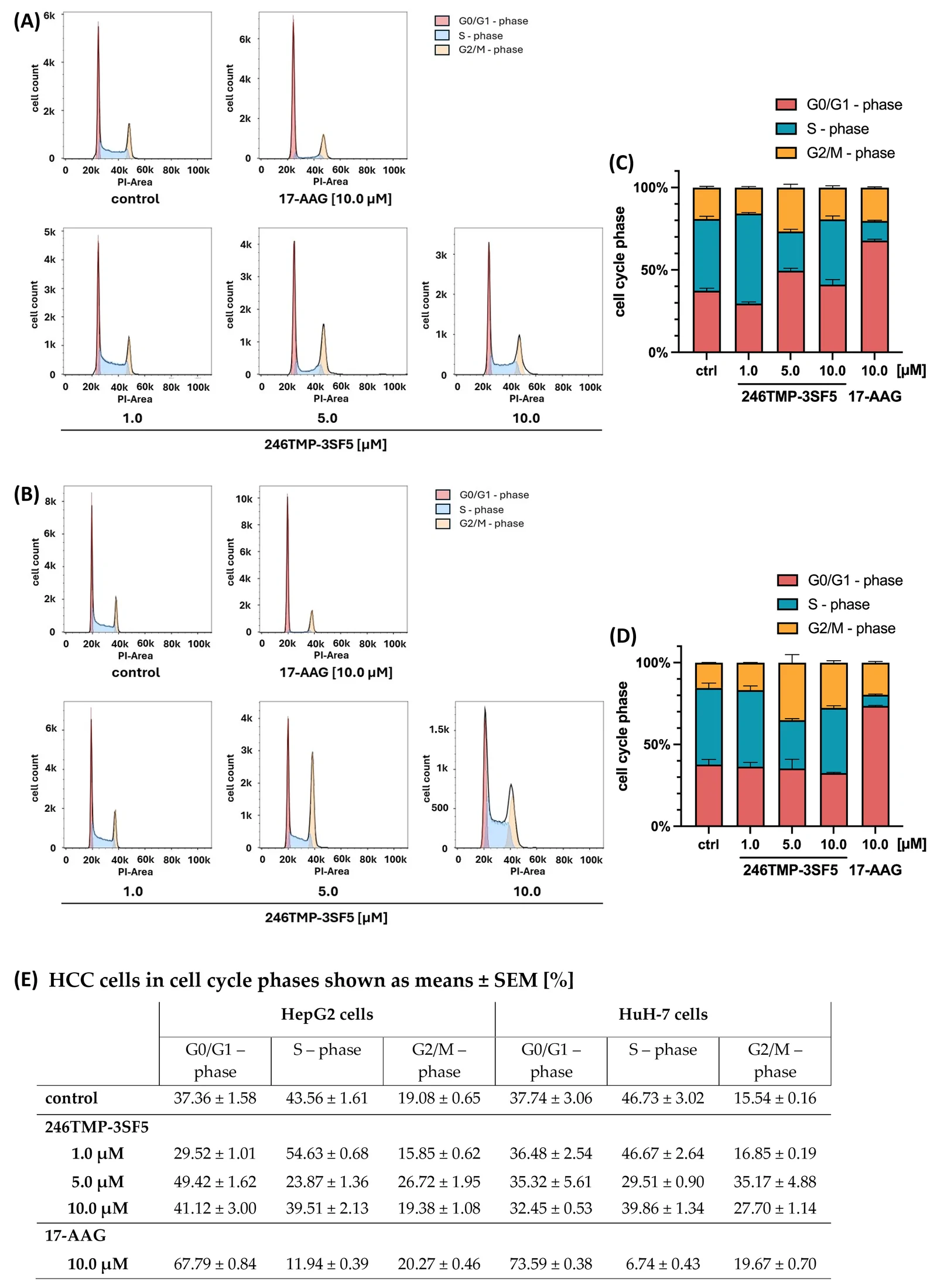
246TMP-3SF5 disrupts the cell cycle of HCC cell lines. Cell cycle analysis revealed changes due to treatment with the novel inhibitor 246TMP-3SF5 in HepG2 (**A**,**C**) and HuH-7 cells (**B**,**D**). *n* = 4 independent experiments were performed. Representative cell cycle histograms (**A**,**B**) and cumulative data of the cell cycle phases as means ± SEM are shown (**C**,**D**). Data is summarized and expressed as means ± SEM in table (**E**).

Cell cycle arresting effects of 246TMP-3SF5 were determined by flow cytometry. Treating HCC cell lines with 246TMP-3SF5 revealed a disruption of the cell cycle compared to control (Figure 6). With increasing concentrations of the novel inhibitor, G0/G1- and G2/M-phase increased. At 1.0 µM 246TMP-3SF5 S-phase was augmented in HepG2 cells (Figure 6A,C,E). Upon treatment of HuH-7 cells with rising concentrations of 246TMP-3SF5, changes in the cell cycle phases were observed, with the G2/M-phase increased compared to control (Figure 6B,D,E). 17-AAG led to a distinct G0/G1-phase arrest in both HCC cell lines, while 246TMP-3SF5 did not cause a clear cell cycle arrest in a specific phase but rather a complex disruption of the cell cycle.

### 3.5. 246TMP-3SF5 Inhibits Cell Migration in HCC Cell Lines

Migration of cells is a hallmark of cancer cells, essential for local invasion of tissue and to form metastases; thus, a novel promising compound inhibiting tumor cell migration would be of particular importance [45]. Anti-migratory effects of 246TMP-3SF5 were investigated through a scratch assay in HCC cell lines; pictures were taken at t = 0 h and t = 24 h after the initial scratch and cell migration rates were assessed through the area of the initial gap filled by migrated HCC cells after 24 h.

**Figure 7:**
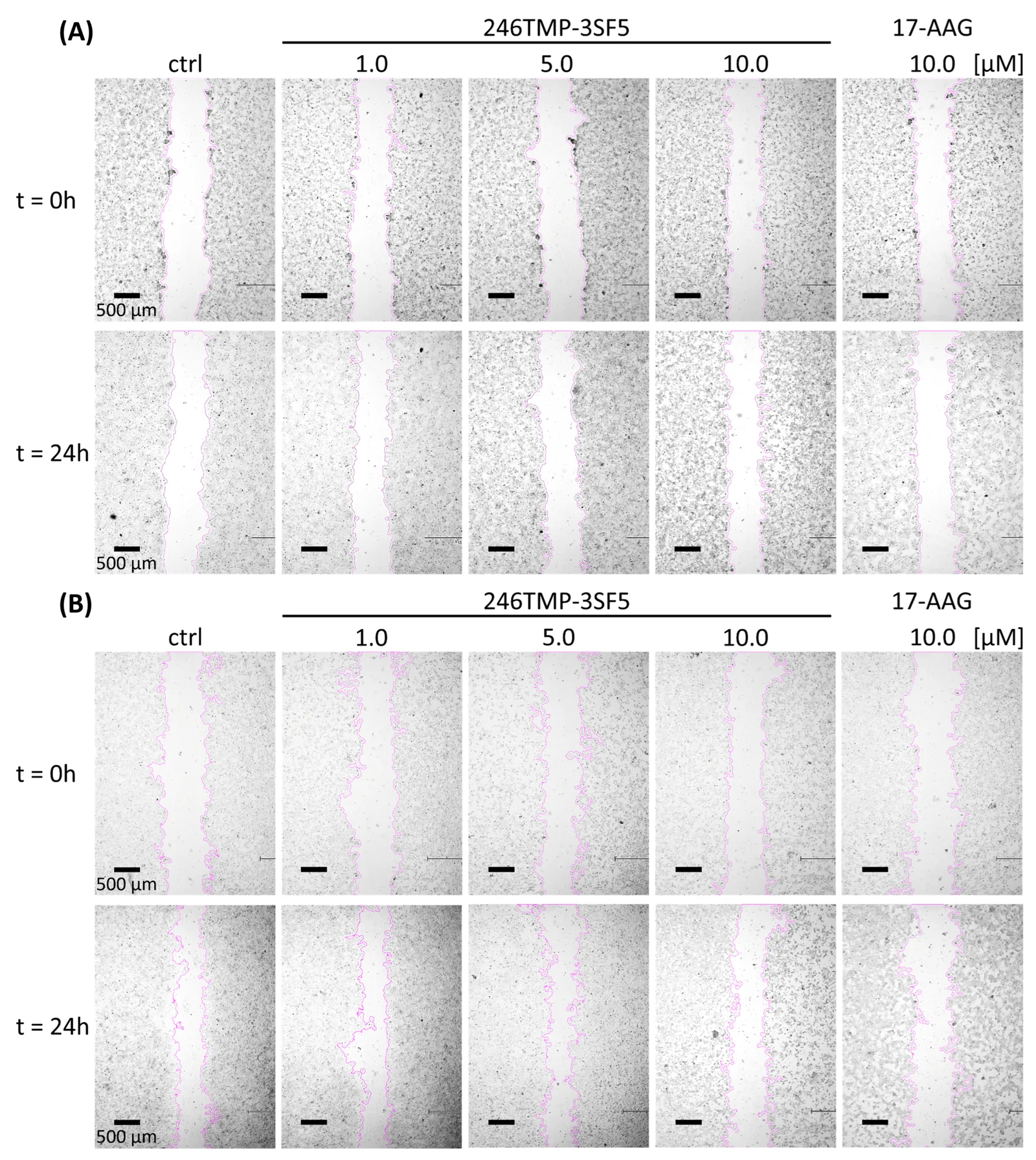

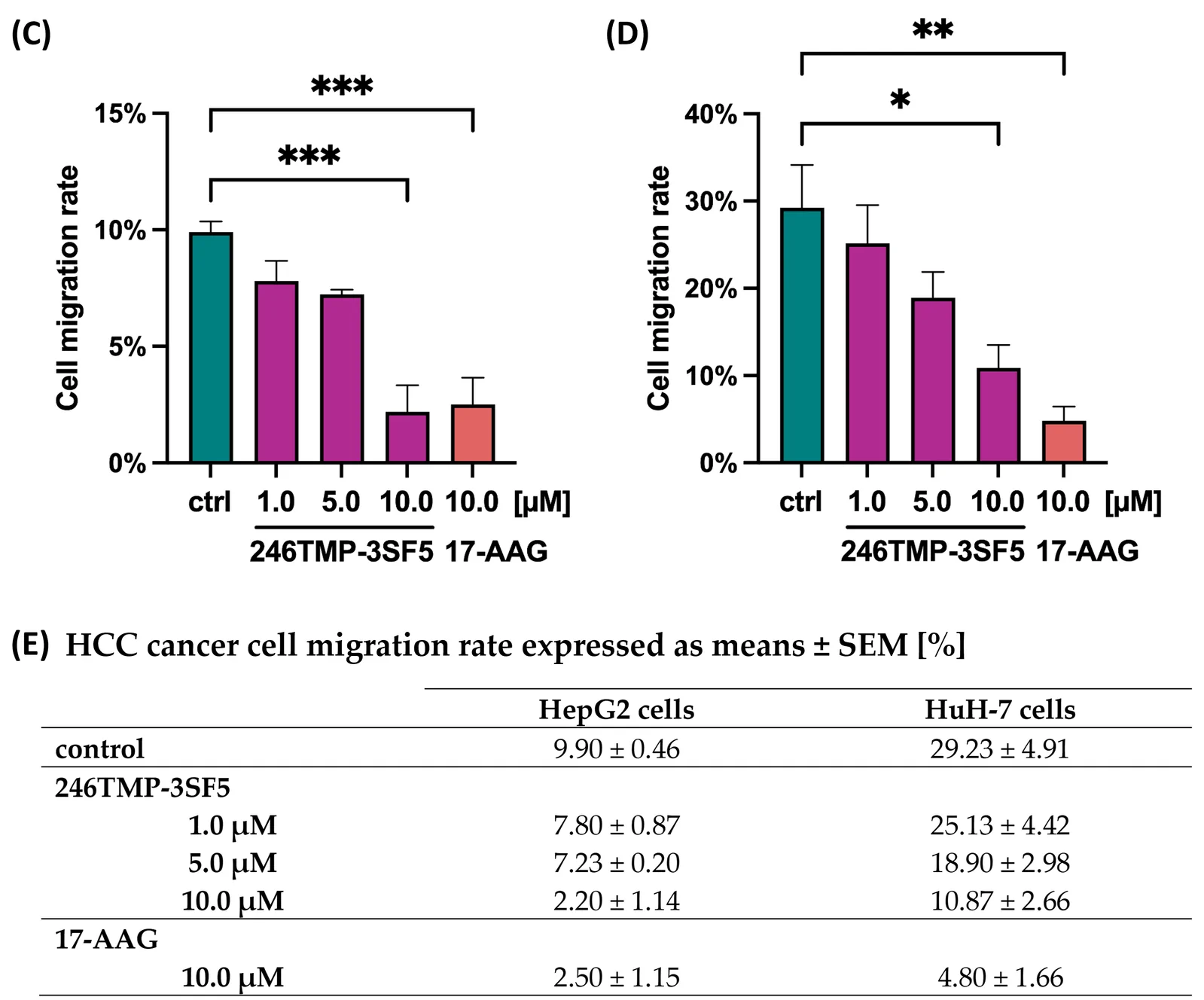
246TMP-3SF5 reduces migration of HCC cells. 246TMP-3SF5 dose-dependently impaired tumor cell migration in HCC cell lines HepG2 (**A**,**C**) and HuH-7 (**B**,**D**). Scratch area was quantified using the wound healing size tool plug-in for ImageJ. Quantified cell migration rate in percentage as means ± SEM is given (**C**,**D**) and values are summarized in the table (**E**). Representative images of *n* = 3 independent experiments are shown. One-way ANOVA followed by Dunnett’s multiple comparisons test was performed and statistical significance is displayed as * *p* ≤ 0.05, ** *p* ≤ 0.01, *** *p* ≤ 0.001.

Following treatment of HCC cell lines with the novel inhibitor 246TMP-3SF5, a dose-dependent decrease in tumor cell migration was observed (Figure 7). In HepG2 cells 10.0 µM 246TMP-3SF5 significantly decreased cell migration, resulting in a migration rate of only 2.2% (±1.1%). The effect was found to be approximately as pronounced as the antimigratory effect of 10.0 µM 17-AAG at a 2.5% (±1.2%) migration rate (Figure 7A,C,E). An even more prominent antimigratory effect of 246TMP-3SF5 was observed in HuH-7 cells, with a decrease in cell migration rate by up to 18.4% (Figure 7B,D,E). Our findings suggest that the novel compound 246TMP-3SF5 impairs tumor cell migration in HCC cell lines, significantly decreasing cancer cell migration rate compared to control.

### 3.6. Anti-Angiogenic Effects of 246TMP-3SF5 In Ovo

Inducing angiogenesis allows tumors to access higher amounts of nutrients and oxygen, sustaining continuous tumor mass growth [45]. Changes in angiogenesis and vascularization of the chorioallantoic membrane (CAM), a highly vascularized membrane in fertilized chicken eggs, were evaluated upon topical application of 246TMP-3SF5.

**Figure 8:**
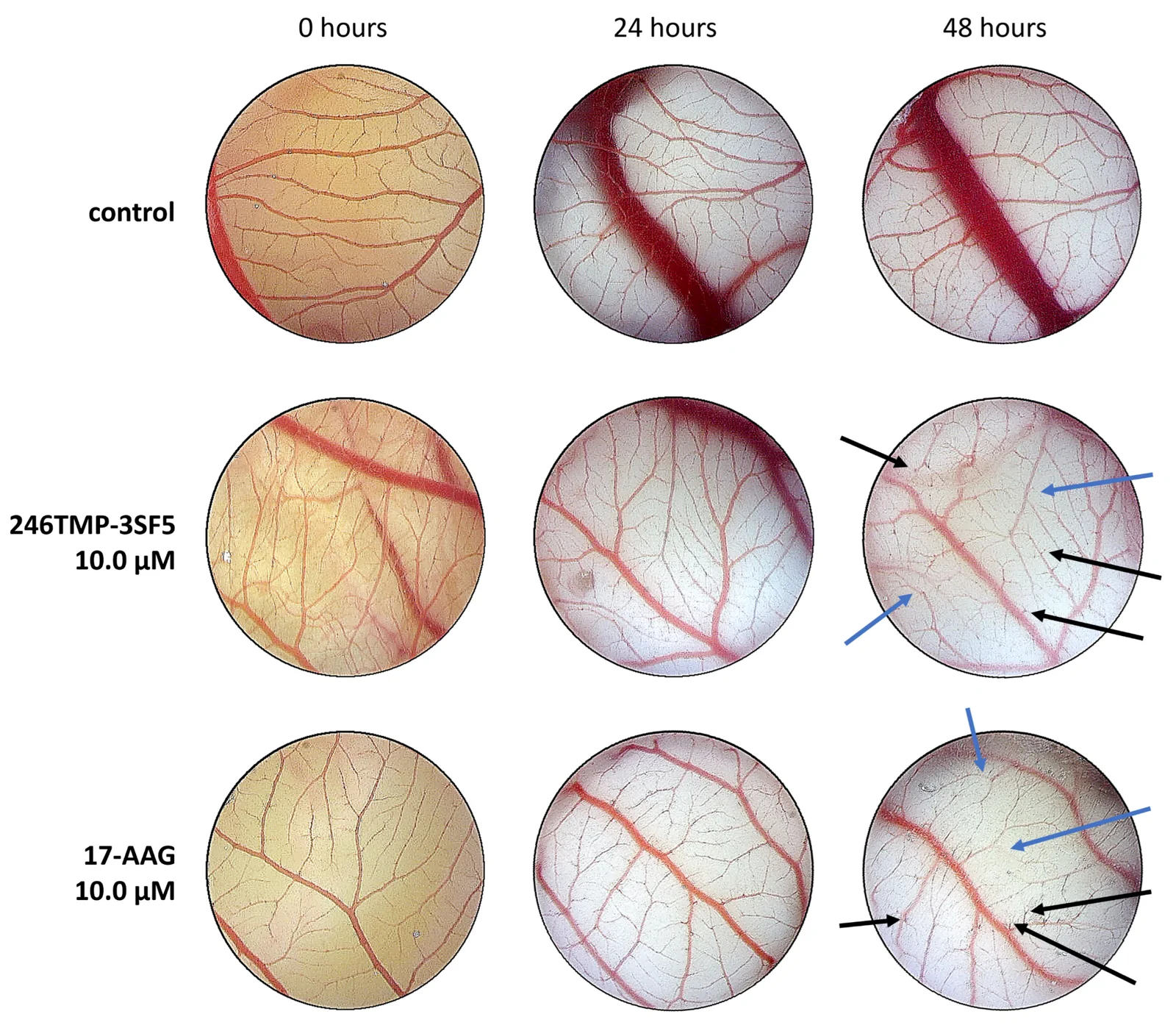
246TMP-3SF5 exhibits anti-angiogenic effects in ovo. 246TMP-3SF5 was topically applied to the CAM of fertilized chicken eggs and changes in angiogenesis and vascular structure were observed over 48 h. At least *n* = 5 eggs for each condition were assessed and representative images are shown. Blue arrows indicate collapsed blood vessels and black arrows indicate altered vessel branching points.

Topical treatment with 10.0 µM 246TMP-3SF5 for up to 48 h caused morphological irregularities of blood vessels of the CAM (Figure 8). Blood vessels collapsed, decreasing in diameter (Figure 8, blue arrows) and vessel branch points appeared as blunt ends of nodule-like structures (Figure 8, black arrows). Overall anti-angiogenic effects of 246TMP-3SF5 became apparent; the vessels of the CAM treated with the novel inhibitor showed a notable reduction of newly formed microvessels and prominent irregularities in the blood vessel network compared to the control. Similar effects were observed for 17-AAG as well. In addition, even at the highest concentration of 10.0 µM of 246TMP-3SF5, no toxicity was observed in chicken embryos during embryonic development days 11 to 13.

#### Morphometric and Fractal Analysis of CAM Vascular Networks

**Figure 9:**
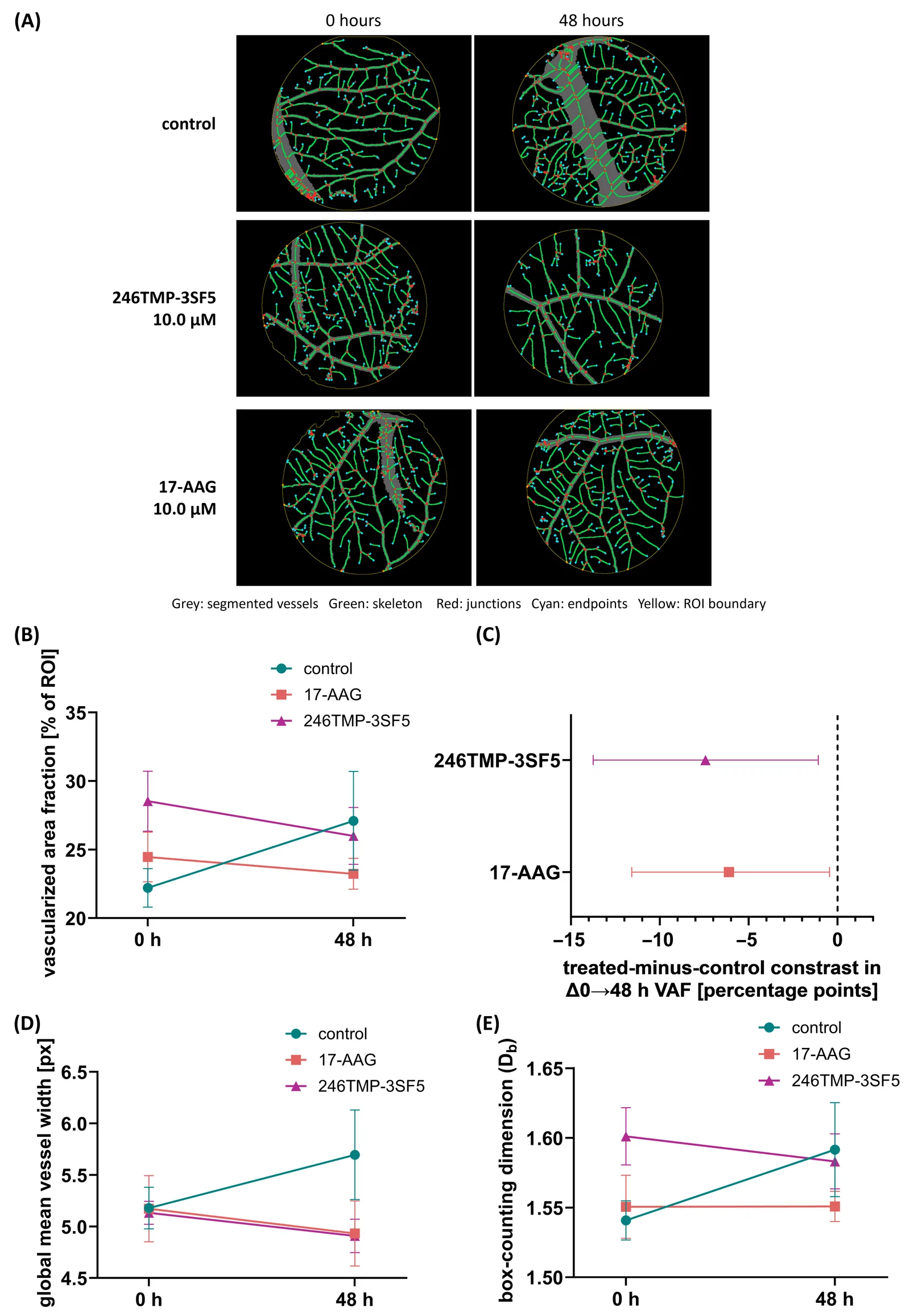
Morphometric and fractal analysis of CAM vascular networks. (**A**) Representative topological overlays at 0 and 48 h with comparable baseline vascularized area fractions are shown. Grey indicates segmented vessel area, green the skeleton, red junction clusters, cyan endpoints and yellow the ROI boundary. Panels (**B**–**E**) summarize all biological samples. (**B**) Group-level vascularized area fraction (VAF, % of ROI) at 0 and 48 h. (**C**) Treated-minus-control contrasts in within-sample 0→48 h VAF change, shown as point estimates with exploratory bootstrap 95% confidence intervals; negative values indicate attenuation of vascularized-area expansion relative to control, and both intervals exclude zero. Group-level changes between 0 and 48 h are additionally shown for (**D**) global mean vessel width (vascular area/skeleton graph length, px) and (**E**) box-counting fractal dimension (D_b_). In panels (**B**,**D**,**E**), means ± SEM are shown (control *n* = 5, 10.0 µM 17-AAG *n* = 5 and 10.0 µM 246TMP-3SF5 *n* = 7).

Quantitative image analysis was performed on 51 vascular masks from 17 biological samples. Vascularized area fraction (VAF) was the clearest discriminator between the treated and control trajectories. Between 0 and 48 h, mean VAF increased in controls from 22.2% to 27.1%, whereas it decreased under 17-AAG from 24.5% to 23.2% and under 246TMP-3SF5 from 28.5% to 26.0% (Figure 9B). For the treated-minus-control contrast in within-sample 0→48 h change, VAF was −6.11 pp under 17-AAG (exploratory bootstrap 95% CI −11.58 to −0.45) and −7.43 pp under 246TMP-3SF5 (95% CI −13.75 to −1.08; Figure 9C).

Global mean vessel width provided the most baseline-balanced morphometric result. Baseline means were virtually identical across groups (control 5.18 px, 17-AAG 5.17 px and 246TMP-3SF5 5.13 px), after which width increased in controls to 5.70 px but decreased under 17-AAG to 4.93 px and under 246TMP-3SF5 to 4.91 px (Figure 9D). Relative to controls, the treated-minus-control contrast in 0→48 h change was −0.755 px under 17-AAG (95% CI −1.394 to −0.164) and −0.739 px under 246TMP-3SF5 (95% CI −1.480 to −0.045). The closely matched baseline values reduce concern that the divergent width trajectories were driven principally by regression to the mean. This area-to-length width estimator therefore provides a quantitative counterpart to the reduction in vessel diameter observed qualitatively in Figure 8. Box-counting dimension (D_b_) increased in controls from 1.541 to 1.592, remained nearly unchanged under 17-AAG (1.550 to 1.551) and decreased under 246TMP-3SF5 (1.601 to 1.583; Figure 9E). The corresponding treated-minus-control contrast was −0.050 for 17-AAG (95% CI −0.100 to +0.005) and −0.068 for 246TMP-3SF5 (95% CI −0.123 to −0.008). Thus, the interval excluded zero for 246TMP-3SF5 but not for 17-AAG. Importantly, baseline values differed between groups for VAF and D_b_, whereas global mean vessel width was nearly identical at baseline across all three groups.

Representative topological overlays illustrate the segmented vascular area, skeleton, junctions and endpoints at 0 and 48 h (Figure 9A).

Taken together, the strongest quantitative CAM signal was attenuation of the normal increase in vascularized area fraction under both inhibitors, accompanied by global vessel narrowing.

### 3.7. Anti-Neoplastic Efficacy of 246TMP-3SF5 In Ovo

**Figure 10:**
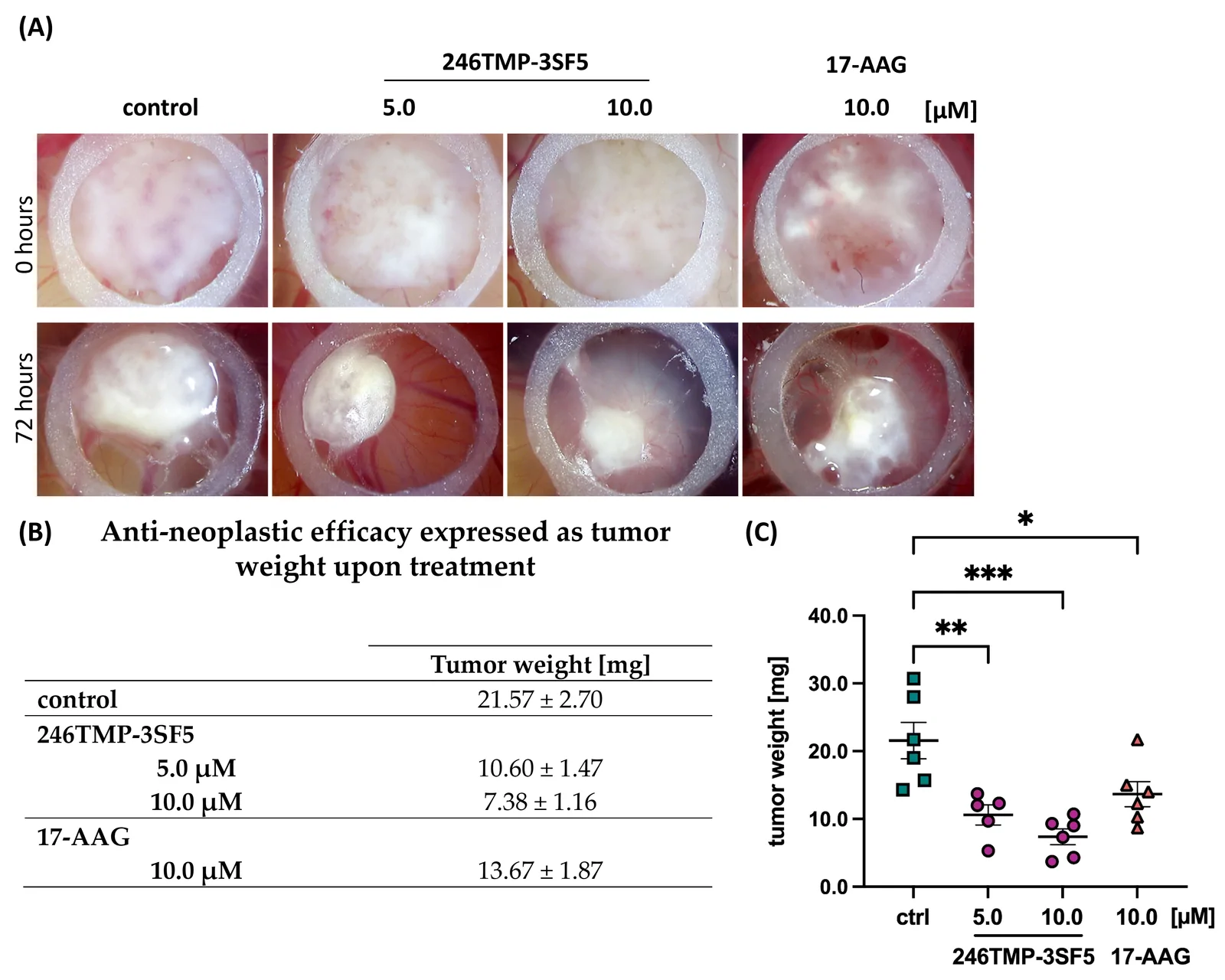
246TMP-3SF5 reduces HCC cell tumor growth in ovo. To investigate the anti-neoplastic effects of 246TMP-3SF5 in ovo, microtumors consisting of HuH-7 cells were placed on the CAM of fertilized chicken eggs and 246TMP-3SF5 or 17-AAG were applied at the indicated concentrations surveilling tumor growth monitored over 72 h (**A**). At least *n* = 5 eggs were analyzed for each condition and representative images are shown. After 72 h tumors were excised and weighed. Data is shown as means ± SEM in table (**B**) and as a scatter plot with mean (**C**). Statistical significance was tested with one-way ANOVA followed by Dunnett’s multiple comparisons test; significance levels displayed as * *p* ≤ 0.05, ** *p* ≤ 0.01, *** *p* ≤ 0.001.

Following previous assessments of the antiproliferative efficacy of 246TMP-3SF5 in HCC cell lines resulting in very promising results as a novel anticancer drug [24], anti-neoplastic efficacy of the novel inhibitor in ovo on fertilized chicken eggs was evaluated. HCC cell line HuH-7-derived microtumors were grown on the CAM of fertilized chicken eggs and treated with 246TMP-3SF5 or 17-AAG. Tumor growth was observed over 72 h and final tumor weight was quantified. 246TMP-3SF5 treatment significantly decreased tumor growth and reduced tumor weight in ovo in a dose-dependent manner compared to untreated controls (Figure 10A–C). Tumor weight dropped to a mean (±SEM) of 10.6 mg (±1.5 mg) at 5.0 µM and 7.4 mg (±1.2 mg) at 10.0 µM 246TMP-3SF5 respectively. The results showed a decrease in tumor growth as well as reduction of tumor weight for 17-AAG as well. However, at 10.0 µM 17-AAG, mean tumor weight amounted to 13.7 mg (±1.9 mg), a similar antineoplastic efficacy observed for 246TMP-3SF5 at only 5.0 µM (Figure 10B). Therefore, the novel inhibitor exceeded the anti-neoplastic efficacy of 17-AAG in ovo (Figure 10C).

### 3.8. Molecular Docking of 246TMP-3SF5 into Heat Shock Protein 90

**Figure 11:**
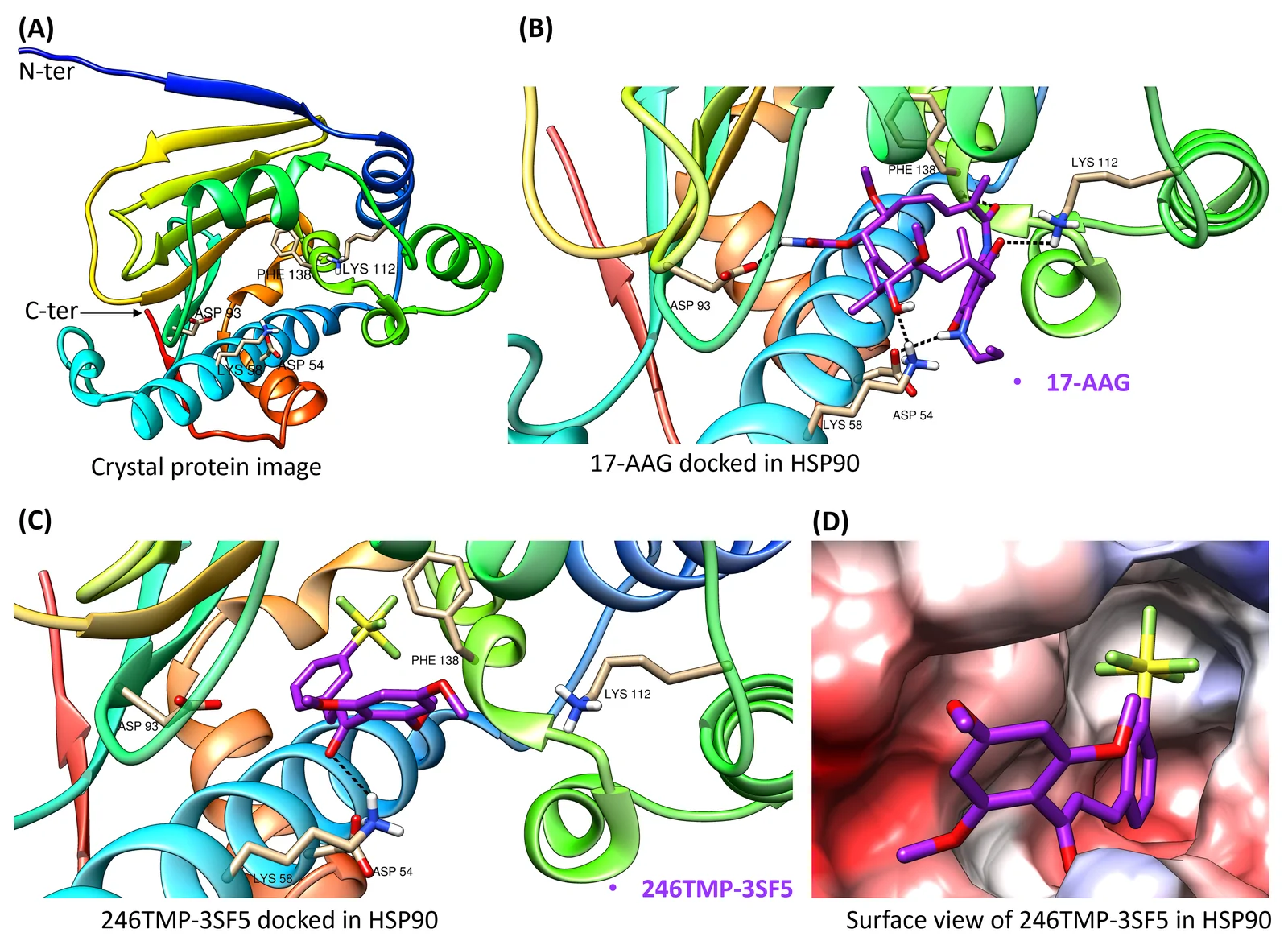
Molecular binding of 246TMP-3SF5 and 17-AAG to N-terminal ATP-binding domain of HSP90. Images show the crystal protein structure of HSP90 (**A**) as well as molecular docking of 17-AAG (**B**) and 246TMP-3SF5 (**C**) in HSP90. Additionally, a surface view of 246TMP-3SF5 in HSP90 is shown in image (**D**). 17-AAG and 246TMP-3SF5 are displayed in purple, while the phenyl–SF_5_ group of 246TMP-3SF5 is shown in yellow-green.

HSP90 is a homodimer molecular chaperone in physiological state, with each subunit containing N-terminal domain (NTD) that contains the ATP/ADP binding site which is crucial for the chaperone’s activity, middle domain (MD) that is involved in interactions with client proteins and co-chaperones and C-terminal domain (CTD) that is responsible for dimerization, forming the interface between the two HSP90 subunits [34] (Figure 11A). It undergoes ATP-driven conformational changes to regulate client protein folding and stability. The docking protocol was first validated by redocking the co-crystallized ligand geldanamycin (GDM), which reproduced key interactions with Asn51, Lys58, Asp93, Lys112, Gly135, and Phe138, yielding a heavy-atom RMSD of 0.202 Å relative to its crystal conformation. As expected, 17-AAG, a semi-synthetic derivative of geldanamycin, adopted a similar binding orientation within the ATP-binding pocket of HSP90 and formed hydrogen-bond interactions with Asp54, Lys58, Asp93, Lys112, and Phe138, with a binding affinity of −9.6 kcal/mol (Figure 11B). The conserved binding mode of GDM and 17-AAG validated the docking protocol and provided a structural basis for evaluating the binding of the newly designed compound 246TMP-3SF5.

Docking of 246TMP-3SF5 revealed a hydrogen-bond interaction with Lys58 and a binding affinity of −7.3 kcal/mol (Figure 11C). Surface visualization showed that the phenyl–SF_5_ moiety was deeply accommodated within a hydrophobic pocket lined by residues such as Met98, Leu107, Val150, and Val186, contributing additional stabilization through favorable hydrophobic (van der Waals) interactions (Figure 11D). To assess the influence of the pentafluorosulfanyl group on binding, the nitro analogue SU086 was also docked into the HSP90 ATP-binding site. SU086 exhibited a comparable binding affinity of −7.3 kcal/mol and formed a hydrogen bond with Phe138; however, it adopted a distinct binding orientation relative to 246TMP-3SF5 (Supplementary Figure S2). Surface analysis indicated that the phenyl–NO_2_ group of SU086 was oriented toward the more polar region of the binding pocket near Asp54 and Ser53, whereas the phenyl–SF_5_ group of 246TMP-3SF5 occupied a predominantly hydrophobic region. The altered binding mode of SU086 is likely attributable to the rigid chalcone framework combined with the different steric and electronic properties of the nitro substituent.

### 3.9. Molecular Dynamics Simulation of 246TMP-3SF5 in Heat Shock Protein 90

**Figure 12:**
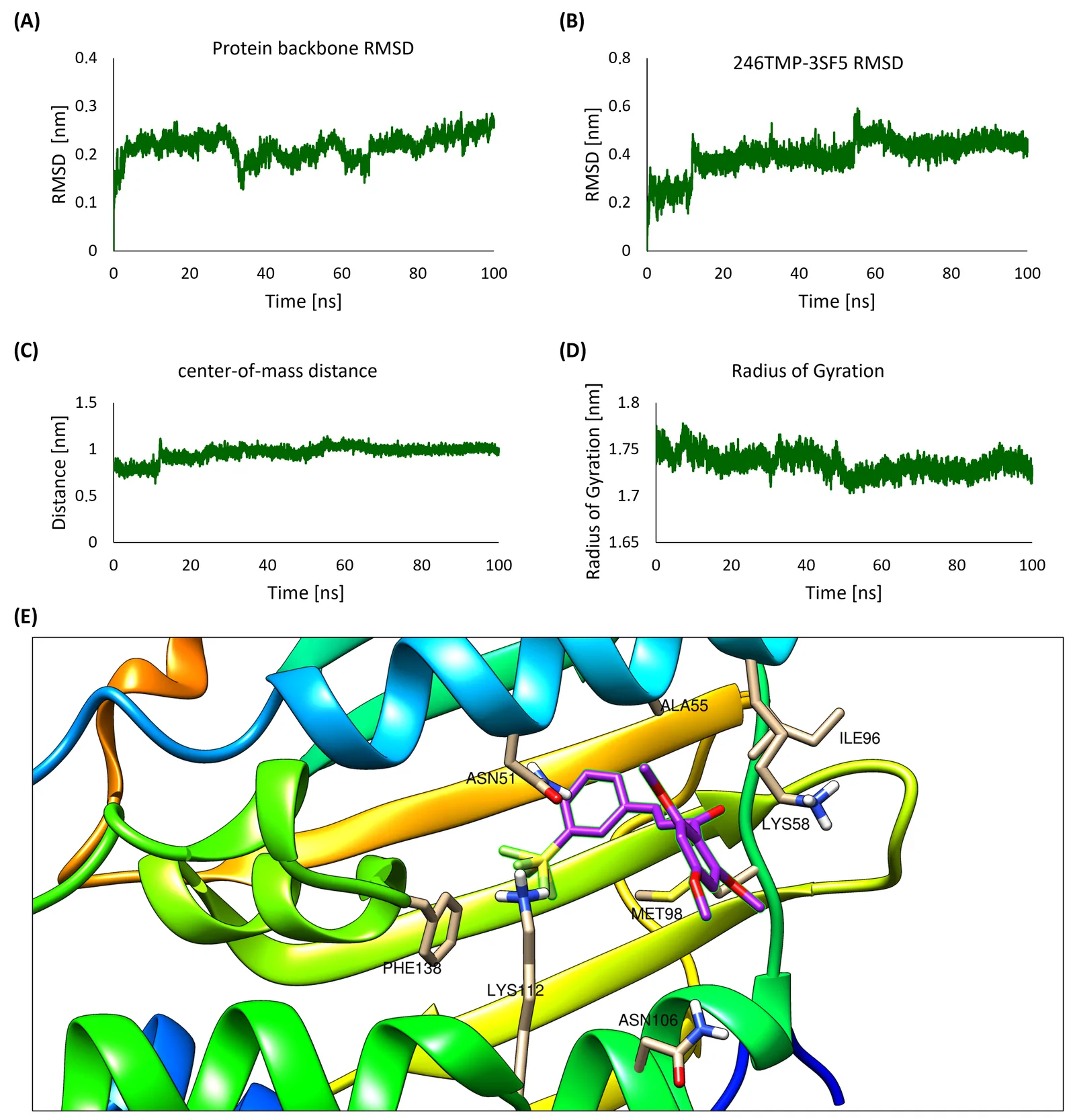
Molecular dynamics simulation of HSP90-246TMP-3SF5 complex. (**A**) HSP90 protein backbone RMSD (**B**) 246TMP-3SF5 ligand RMSD (**C**) Protein–ligand center-of-mass distance (**D**) Radius of gyration (**E**) Last frame of 100 ns simulation.

The structural stability of the HSP90–246TMP-3SF5 complex was assessed by monitoring the root mean square deviation (RMSD) of the protein backbone and ligand throughout the molecular dynamics simulation. The backbone remained structurally stable with average RMSD of 0.21 nm, indicating that the overall protein structure was well maintained during the simulation (Figure 12A) and the overall structure of protein remained intact which can be seen from RMSF plot (Supplementary Figure S3). Similarly, the ligand demonstrated stable binding in the ATP-binding pocket with an average RMSD of 0.39 nm, suggesting that 246TMP-3SF5 retained a consistent binding orientation within the ATP-binding pocket of HSP90 (Figure 12B) and an average protein–ligand center-of-mass distance of 0.96 ± 0.07 nm, suggesting that the ligand remained firmly bound without evidence of dissociation (Figure 12C). The structural integrity of HSP90 was further confirmed by the radius of gyration and solvent-accessible surface area analyses. The radius of gyration remained nearly constant throughout the simulation with an average value of 1.735 ± 0.011 nm, indicating preservation of the compact protein fold (Figure 12D). The last frame of the simulation shows 246TMP-3SF5 properly occupying the ATP-binding site, with the -SF_5_ moiety in hydrophobic pocket of the site (Figure 12E). Similarly, the solvent-accessible surface area fluctuated only minimally around 113.09 ± 2.13 nm^2^, demonstrating that ligand binding did not induce significant structural expansion or collapse of the protein.

Hydrogen bond analysis showed only transient direct hydrogen-bond interactions between 246TMP-3SF5 and residues within the ATP-binding pocket, with an average occupancy of 0.014 hydrogen bonds per frame. Although persistent hydrogen bonds were not observed, the ligand remained stably associated with HSP90 throughout the simulation, indicating that binding is predominantly stabilized by non-covalent interactions rather than by an extensive hydrogen-bonding network. Protein–ligand interaction energy analysis further supported stable complex formation. The average short-range Coulombic and Lennard-Jones interaction energies were −157.97 kJ/mol and −133.54 kJ/mol, respectively, demonstrating that both electrostatic and van der Waals interactions contribute substantially to ligand stabilization within the ATP-binding pocket.

**Figure 13:**
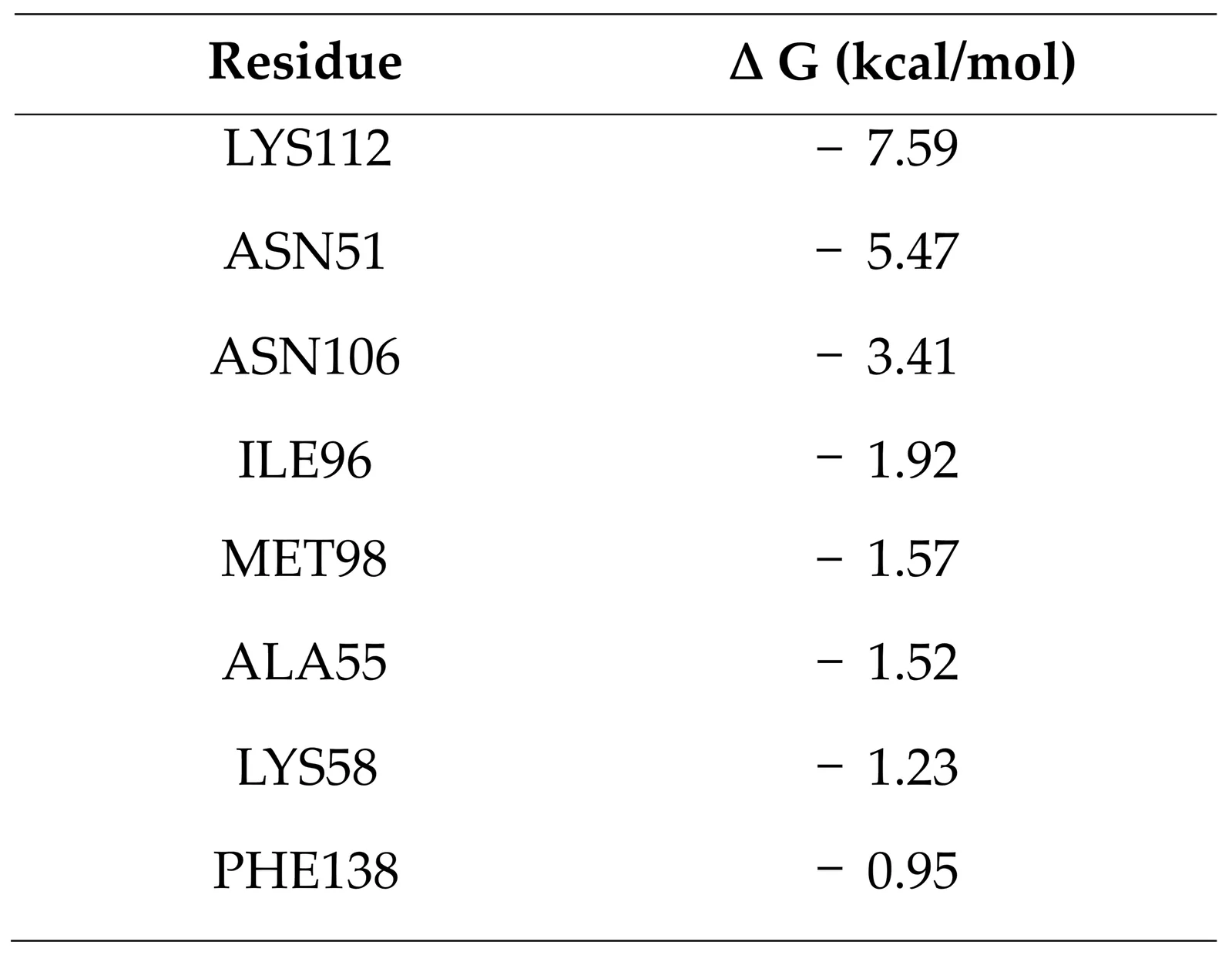
Per-residue energy decomposition. The most contributing residues to the binding of 246TMP-3SF5 to HSP90.

Quantitatively, the binding energy was predicted by MM/GBSA (Molecular Mechanics/Generalized Born Surface Area) calculations over 501 trajectory frames. The calculated binding free energy (ΔG_bind) (calculated as ΔG_bind = G_complex − (G_receptor + G_ligand)) was −29.57 ± 13.99 kcal/mol, indicating thermodynamically favorable binding of 246TMP-3SF5 to HSP90. The favorable binding free energy primarily resulted from strong gas-phase interactions (ΔG_GAS = −117.24 kcal/mol), which were partially offset by the unfavorable solvation contribution (ΔG_SOLV = 87.67 kcal/mol), yielding an overall negative binding free energy consistent with stable complex formation. Per-residue free-energy decomposition identified several residues within the ATP-binding pocket as major contributors to ligand stabilization. Among these, LYS112 exhibited the largest favorable contribution, followed by ASN51, ASN106, ILE96, MET98, ALA55, and LYS58, indicating that both electrostatic and hydrophobic interactions cooperate to stabilize 246TMP-3SF5 within the binding cavity (Figure 13).

Collectively, the molecular docking, molecular dynamics simulation, interaction energy analysis, and MM/GBSA calculations consistently demonstrate that 246TMP-3SF5 forms a stable complex with the ATP-binding pocket of HSP90. These computational findings support the proposed mechanism of HSP90 inhibition and complement the observed antiproliferative and pro-apoptotic activities of the compound in hepatocellular carcinoma models.

## 4. Discussion

HSP90 is a promising therapeutic target in HCC treatment as it stabilizes various client proteins involved in hepatocarcinogenesis and is often overexpressed, correlates with advanced tumor stage and poor prognosis [46,47]. Antiproliferative assessment of the novel pentafluorosulfanyl-substituted chalcone 246TMP-3SF5, derived from the HSP90 inhibitor SU086, revealed pronounced time- and dose-dependent antiproliferative effects against HCC cell lines with low micromolecular IC_50_ values at 1.3 (±0.2 µM) for HepG2 and 2.2 (±0.3 µM) for HuH-7 cells. Interestingly, previous assessments revealed that the parent drug SU086 was not effective in HCC cell models, with IC_50_ values above 10.0 µM [24]. The data suggests the modification to the original chalcone structure positively impacted the biological antiproliferative activity of 246TMP-3SF5 against HepG2 and HuH-7 cells. In comparison 17-AAG achieved similar IC_50_ values in HepG2 cells, while being more effective in HuH-7 cells. It is noteworthy that the novel inhibitor reached lower IC_50_ values compared to sorafenib, which is currently used in first-line treatment of advanced-stage HCC. Investigation regarding the selectivity of the antiproliferative effect observed against HCC cells revealed a selectivity index of >1 for 246TMP-3SF5, while being below 1 for 17-AAG. These results indicate that the novel inhibitor exerts selective antiproliferative effects greater in HCC cell lines than in non-malignant cells. Moreover, 246TMP-3SF5 also significantly reduced HCC microtumor mass in ovo at 5.0 and 10.0 µM by approximately 50% compared to control, confirming great growth suppressing effects.

The investigation of the mode of action of 246TMP-3SF5 revealed a multitude of effects in liver cancer cell models. Investigating apoptosis a significant dose-dependent increase in cells in the sub-G1 peak, significant elevation of apoptosis key caspase-3 activity and cleavage of PARP were observed, indicating apoptosis to be a relevant mode of cell death caused by treatment of HCC cells with 246TMP-3SF5. It is noteworthy that although the HSP90 reference inhibitor 17-AAG also showed an increase in the sub-G1 peak and elevation of caspase-3 activity, the effect at an equal concentration of 10.0 µM compared to 246TMP-3SF5 was not as prominent. Preclinical studies have shown 17-AAG to induce apoptosis; however, the described pathways differ from the ones assessed in our study. 17-AAG seems to induce apoptosis through degradation of multiple HSP90 client proteins, mainly via the intrinsic mitochondrial pathway, although differences were reported depending on the cell line that was assessed [48,49].

Additionally, our findings revealed increased intracellular ROS levels, significant GSH depletion, as well as significant dose-dependent MDA concentration elevation, suggesting ferroptosis occurred upon treatment with 246TMP-3SF5. Although 10.0 µM 17-AAG also reduced intracellular GSH levels, the effect is significantly stronger for 246TMP-3SF5 at equal concentration. Notably, 17-AAG causes almost no increase in MDA concentration upon treatment. The observed GSH depletion and rise in MDA concentration due to treatment with 246TMP-3SF5 and the ferroptosis inducer erastin were similarly pronounced. This hints at a difference between 246TMP-3SF5’s and 17-AAG’s ability to induce ferroptosis. 17-AAG has been shown to react chemically with GSH, forming adducts depleting intracellular GSH, in line with our findings [50]. Although GSH is an essential cofactor for GPX4, part of the Xc^−^/GSH/GPX4 system, to date no study has demonstrated that 17-AAG directly induces ferroptosis in HCC cells, consistent with our findings. On the contrary, our results indicate that 246TMP-3SF5 induces ferroptosis in HCC cell lines.

Further investigations revealed that 246TMP-3SF5 induced cell cycle disruption, although no clear cell cycle arrest was observed. In HuH-7 cells an increase in the G2/M-phase upon treatment with 246TMP-3SF5 was noticeable. In contrast, 17-AAG caused HCC cell arrest in the G0/G1 phase of the cell cycle. Other studies also found 17-AAG to cause cell cycle arrest. Previously, an increase in HuH-7 cells in the G0/G1 phase up to 55.9% (±7.1%), alongside a G2/M phase arrest with 28.9% (±13.0%), was observed, which the authors explained by cdc2 degradation [51].

HCCs have been shown to be highly vascularized tumors, with HSP90 overexpression promoting angiogenesis; thus, a drug exhibiting antiangiogenic effects is of particular interest [18,46,47]. Our results show that 246TMP-3SF5 exerts significant anti-migratory as well as anti-angiogenic effects in ovo. Quantitative morphometry corroborated the qualitative CAM observations. The clearest effect was on vascularized area fraction: controls increased over 48 h, whereas both treated groups decreased, and the treated-minus-control contrasts in within-sample change excluded zero. Both treatments also reversed the normal increase in global mean vessel width, providing a direct quantitative counterpart to the collapsed and narrowed vessels indicated by the blue arrows in Figure 8. Because baseline vessel width was virtually identical across groups (5.18, 5.17 and 5.13 px), the divergent width trajectories are less vulnerable than VAF or box-counting dimension to regression to the mean and constitute the most internally robust morphometric result. 246TMP-3SF5 additionally reduced box-counting dimension relative to controls, consistent with reduced multiscale occupation of the vascular network, whereas the interval for 17-AAG included zero. Moreover, molecular docking into HSP90 demonstrated the phenyl-SF_5_ moiety stably accommodated within a hydrophobic pocket of the ATP-binding site. Molecular dynamics, interaction energy analysis and MM/GBSA calculations consistently suggest that 246TMP-3SF5 stably binds to the ATP-binding pocket of HSP90, revealing the likely form of interaction of the novel inhibitor with HSP90. Inhibition of HSP90 leads to proteasomal degradation of its client proteins, many of which are of high importance for angiogenesis and migration of tumor cells. HSP90 inhibitors impair multiple pro-angiogenic pathways, such as reducing the expression of VEGF receptors on endothelial cells. Additionally, crucial for angiogenesis, HIF-1α is also part of the long list of HSP90 client proteins. Amongst others it drives VEGF transcription under hypoxic conditions, allowing for tumor angiogenesis. Through HIF-1α degradation, inhibition of HSP90 leads to impaired endothelial recruitment and angiogenesis, inhibiting a crucial pro-angiogenic pathway of tumor cells [52,53,54,55]. Similarly, HSP90 inhibitors impair tumor cell migration by causing proteasomal degradation of important receptor tyrosine kinases, such as EphA2, which is involved in cellular motility and invasiveness [56,57,58,59,60]. Additionally, a fraction of HSP90α is secreted into the extracellular space, playing an important role in cell migration, cytoskeletal reorganization, as well as extracellular matrix remodeling.

Many different HSP90 inhibitors have been developed and preclinically as well as clinically evaluated against various cancer entities. Some examples evaluated in HCC cell models include geldanamycin derivatives 17-AAG, 17-DMAG, as well as resorcinol-based inhibitor AUY922, non-quinone inhibitor PU-H71, SNX-2112 and STA-9090 [47,61,62,63,64]. Often preclinical evaluations reveal promising effects against various cancers; however, clinical translation remains highly challenging. Thus far pimitespib is the only HSP90 inhibitor approved for clinical treatment [65]. It received official approval for the treatment of gastrointestinal stromal tumor patients refractory to tyrosine kinase inhibitor therapy in Japan in 2022 [66,67,68]. To this day no HSP90 inhibitor has been approved for treatment of HCC. Main challenges in clinical trials are dose-limiting toxicities, with hepatotoxicity most relevant in HCC treatment where liver function is often already impaired. For geldanamycin derivatives such as 17-AAG, the benzoquinone moiety revealed itself to be central to the hepatotoxicity observed. Patients in clinical trials showed dose-limiting hepatotoxicity through aminotransferase elevation [69,70,71]. In contrast 246TMP-3SF5 is derived from the HSP90 inhibitor SU086, containing a chalcone backbone and previous investigations showed no increased toxicity in HCC cell models, even at high concentrations of the drug [24]. Representing an in vivo setting, our in ovo experiments showed no increased mortality of chicken embryos, suggesting 246TMP-3SF5 exhibits no remarkable toxicity; however, further in vivo experiments evaluating toxicity and specifically hepatotoxicity are needed.

Regarding the clinical application of HSP90 inhibitors, combining HSP90 inhibitors with targeted therapies, immune checkpoint inhibitors or chemotherapy has shown synergistic effects, improving anticancer activity. Moreover, combination therapy allows for the decrease of the dose of each drug, therefore also reducing individual drug-associated toxicity. Various combinations are currently being investigated in clinical trials, with some reporting improved clinical response to treatment [66,72]. Another interesting strategy being investigated is combining HSP90 inhibitor therapy with heat stress through thermal therapy. Previously, studies have shown combining hyperthermia with other treatment modalities to be beneficial [73]. Preclinical evaluation showed HSP90 inhibitor STA-9090 sensitizes HCC to hyperthermia-induced DNA damage [63]. Therefore, investigating how the combination of 246TMP-3SF5 with other drugs used in HCC treatment or hyperthermia can impact the anticancer activity is of great interest.

## 5. Conclusions

In summary, our findings in this study provide important insight into the mechanisms of action of the promising novel inhibitor 246TMP-3SF5. Our results indicate that apoptosis and ferroptosis play an important role in the pronounced antiproliferative effects observed. Following treatment cell cycle disruption in both HCC cell lines was observed and migration of cancer cells and induction of angiogenesis were significantly impaired. Analysis of CAM vascular networks indicated that the normal increase in vascularized area fraction was attenuated and was accompanied by global vessel narrowing. Additionally, our results indicate a significant treatment-induced reduction of microtumor growth in ovo, while investigation of compound selectivity revealed the novel inhibitor to be more effective in HCC cells and therefore show selectively for cancer cells. Lastly, our findings suggest the novel inhibitor interacts with HSP90 by binding to its ATP-binding pocket. Overall, we found that 246TMP-3SF5 addresses many hallmarks of cancer essential for tumor growth and spread, thus being a promising novel compound meriting further investigations and evaluation as a new anticancer drug in the treatment of hepatocellular carcinoma.

## Data Availability

Original data of CAM analysis, the 51 binary vascular masks, the analysis code and the per-sample morphometric and fractal measurements are available through this Google Drive depository: https://drive.google.com/file/d/1XFhFHNuPKxZNEL8exkfiakgv-jykOxWG/view?usp=sharing (accessed on 29 July 2026).

## Supplementary Materials

The following supporting information can be downloaded at https://www.mdpi.com/article/10.3390/cancers18162640/s1, File S1: The original blot images. Figure S1: Antiproliferative effects of SU086, 17-AAG and sorafenib on HCC cell lines. Antiproliferative effects were assessed through crystal violet staining and dose-response curves were generated for SU086 (in HepG2 cells (A), in HuH-7 cells (B)), 17-AAG (in HepG2 cells (C), in HuH-7 cells (D)) and sorafenib (in HepG2 cells (E), in HuH-7 cells (F)). Data is shown as means ± SEM and curves were generated through non-linear regression analysis. *n* ≥ 3 independent experiments were performed in triplicate or more. Figure S2: Molecular binding of SU086 to HSP90. (A) SU086, a nitro derivative of 246TMP-3SF5, when docked in HSP90, shows different binding mode compared to 246TMP-3SF5. It has an affinity score of −7.3 kcal/mol. (B) SU086 docked in HSP90 for reference to show the nitro group flipped from hydrophobic pocket to hydrophlic pocket. SU086 shows interaction with Phe138. (C) The surface view of SU086 shows phenyl–NO_2_ group is facing towards Asp54 and Ser53, which gives polarity to the local area, giving stability to the molecule. In the case of 246TMP-3SF5, the –SF5 group was facing upwards, where all hydrophobic groups are residing like Met98, Leu107, Val150 and Val186. Owing to the rigid conformation of chalcones, there is change in binding mode of SU086. Figure S3: RMSF of protein backbone during MD simulation. The root mean square fluctuations show high fluctuations in N-terminal and C-terminal loops of the protein, while α-helices and β-sheets do not show any major fluctuations, indicating that the secondary structure of the protein remains undistorted during simulation.

## Abbreviations

The following abbreviations are used in this manuscript:

HCC	Hepatocellular carcinoma
BCLC	Barcelona Clinic Liver Cancer
HSP	Heat shock protein
PARP	Poly-(ADP-ribose)-polymerase
ROS	Reactive oxygen species
GSH	Glutathione
MDA	Malondialdehyde
CAM	Chorioallantoic membrane
HSP90	Heat shock protein 90
GDM	Geldanamycin
RMSD	Root mean square deviation
SI	Selectivity Index
VAF	Vascularized area fraction
D_b_	Box-counting fractal dimension
ROI	Region of interest
CI	Confidence interval
pp	Percentage points
