# Supplementary material for: Characterization of the New Pentafluorosulfanyl-Substituted Chalcone 246TMP-3SF5 as a Potential New Treatment Option Against Hepatocellular Carcinoma
Source: Cancers (Basel). 2026 Aug 16;18(16):2640. doi: 10.3390/cancers18162640 (PMC13510392; doi:10.3390/cancers18162640)
Supplement: Supplementary file 1 [file cancers-18-02640-s001.zip › cancers-4404814-File S1.pdf]

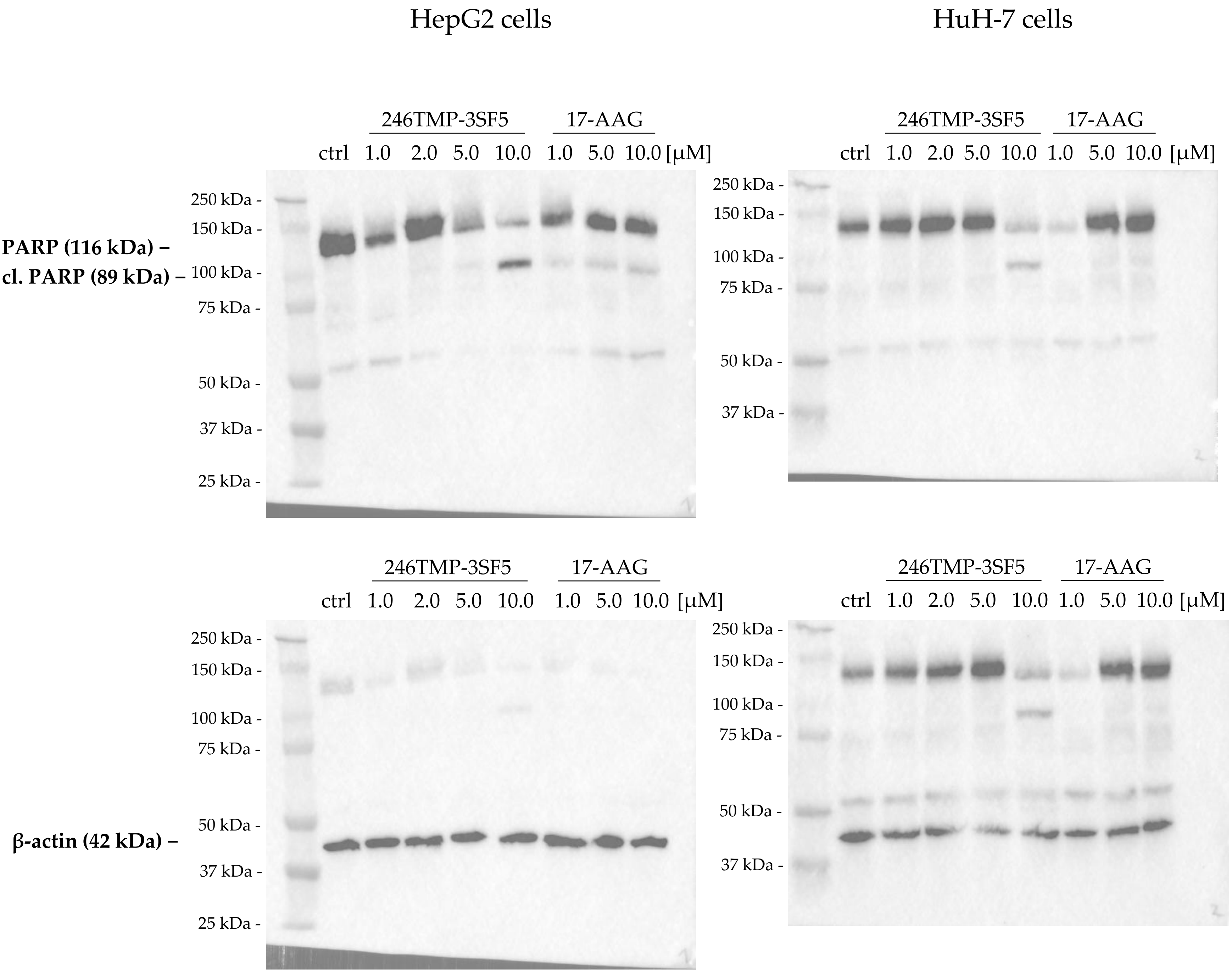
HepG2 cells
HuH-7 cells
246TMP-3SF5
17-AAG
ctrl 1.0 2.0 5.0 10.0 1.0 5.0 10.0 [μM]
250 kDa -
150 kDa -
100 kDa -
75 kDa -
50 kDa -
37 kDa -
25 kDa -
PARP (116 kDa) –
cl. PARP (89 kDa) –
β-actin (42 kDa) –

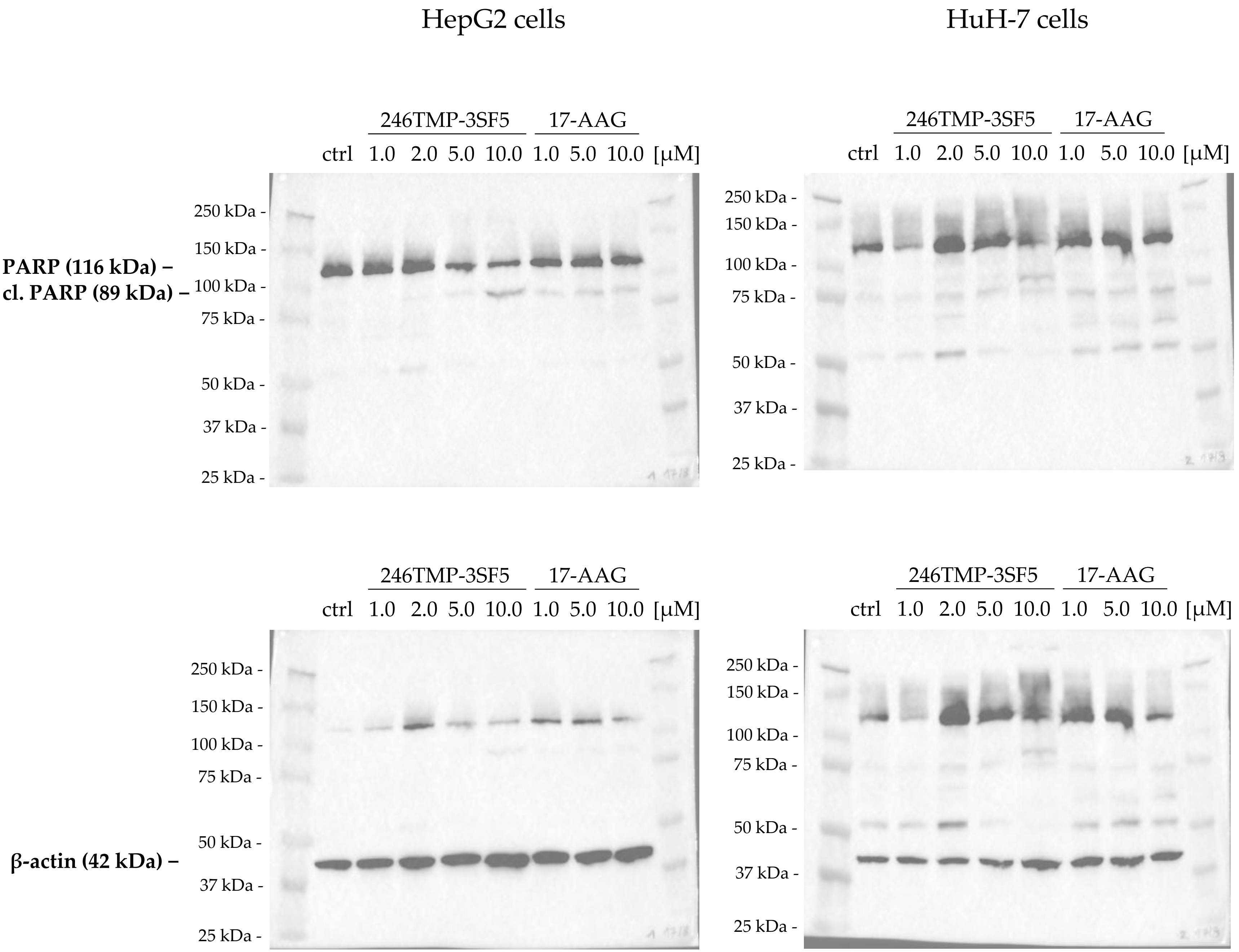

HepG2 cells
HuH-7 cells
246TMP-3SF5
17-AAG
ctrl 1.0 2.0 5.0 10.0 1.0 5.0 10.0 [μM]
250 kDa -
150 kDa -
100 kDa -
75 kDa -
50 kDa -
37 kDa -
25 kDa -
PARP (116 kDa) –
cl. PARP (89 kDa) –
β-actin (42 kDa) –

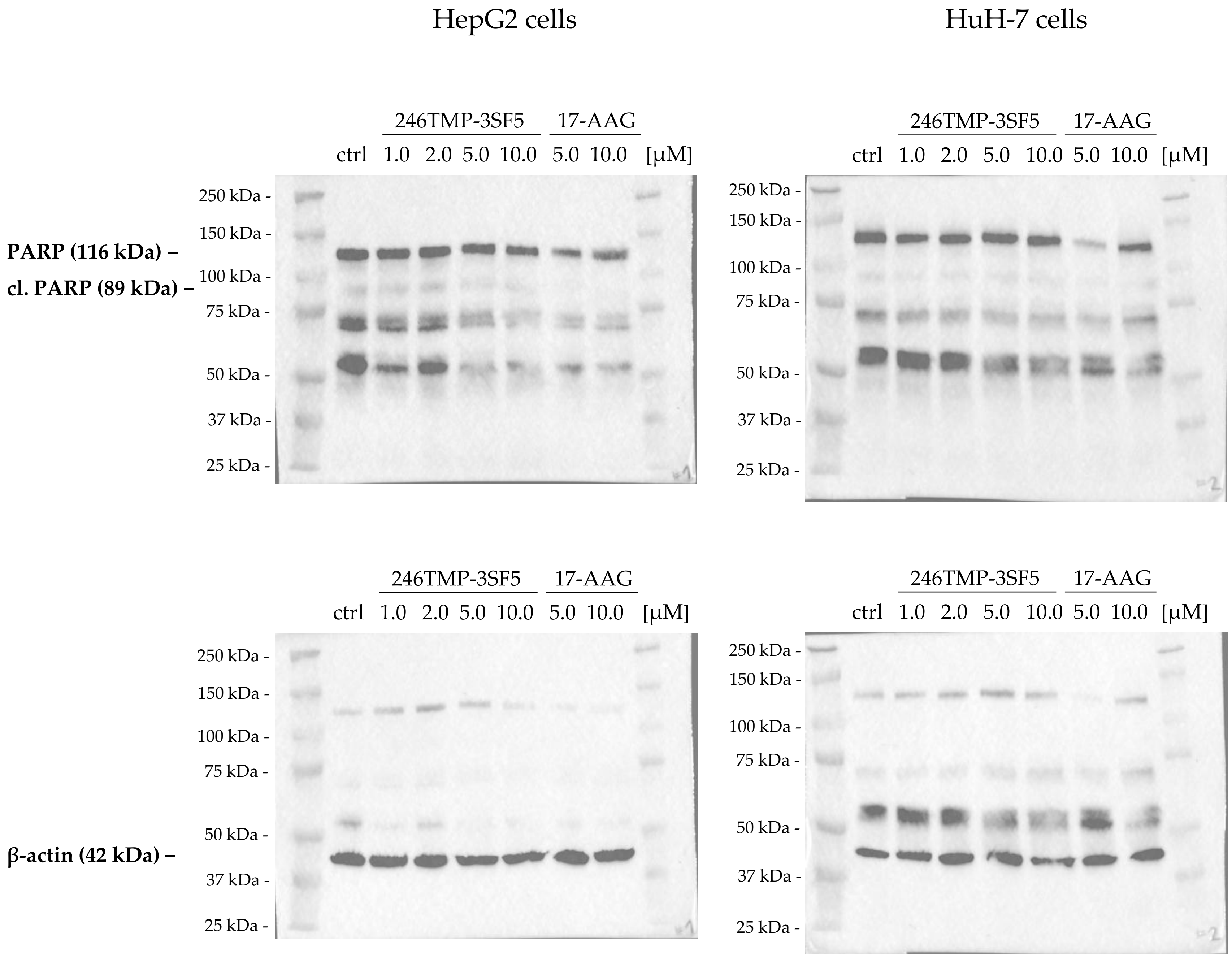
HepG2 cells
HuH-7 cells
PARP (116 kDa) –
cl. PARP (89 kDa) –
β-actin (42 kDa) –
246TMP-3SF5
17-AAG
ctrl 1.0 2.0 5.0 10.0 5.0 10.0 [μM]
250 kDa -
150 kDa -
100 kDa -
75 kDa -
50 kDa -
37 kDa -
25 kDa -
