# Supplementary material for: Characterization of the New Pentafluorosulfanyl-Substituted Chalcone 246TMP-3SF5 as a Potential New Treatment Option Against Hepatocellular Carcinoma
Source: Cancers (Basel). 2026 Aug 16;18(16):2640. doi: 10.3390/cancers18162640 (PMC13510392; doi:10.3390/cancers18162640)
Supplement: Supplementary file 1 [file cancers-18-02640-s001.zip › cancers-4404814-supplementary-english.pdf]

Supplementary Materials: Characterization of the New Pentafluorosulfanyl-Substituted Chalcone 246TMP-3SF5 as a Potential New Treatment Option Against Hepatocellular Carcinoma

Alessandra Viperino, Linda Hammerich, Bernhard Biersack, Supriya Pradhan, Ion Andronache, Isabel Groth,
Nicole Edel, Michael Höpfner and Bianca Nitzsche

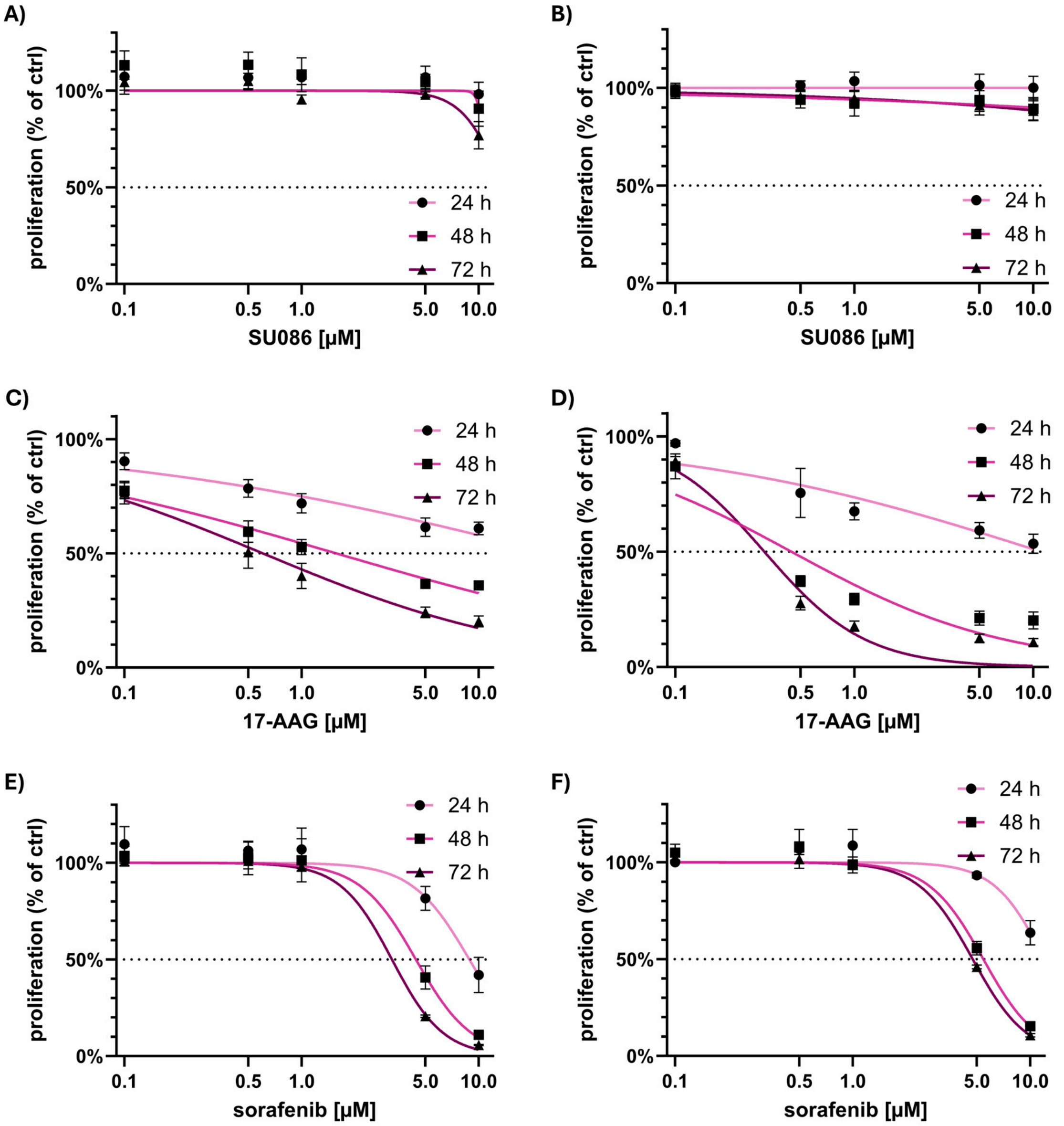

Figure S1. Antiproliferative effects of SU086, 17-AAG and sorafenib on HCC cell lines.

Antiproliferative effects were assessed through crystal violet staining and dose-response curves were generated for SU086 (in HepG2 cells (**A**), in HuH-7 cells (**B**)), 17-AAG (in HepG2 cells (**C**), in HuH-7 cells (**D**)) and sorafenib (in HepG2 cells (**E**), in HuH-7 cells (**F**)). Data is shown as means ± SEM and curves were

generated through non-linear regression analysis. $n \geq 3$ independent experiments were performed in triplicate or more.

A

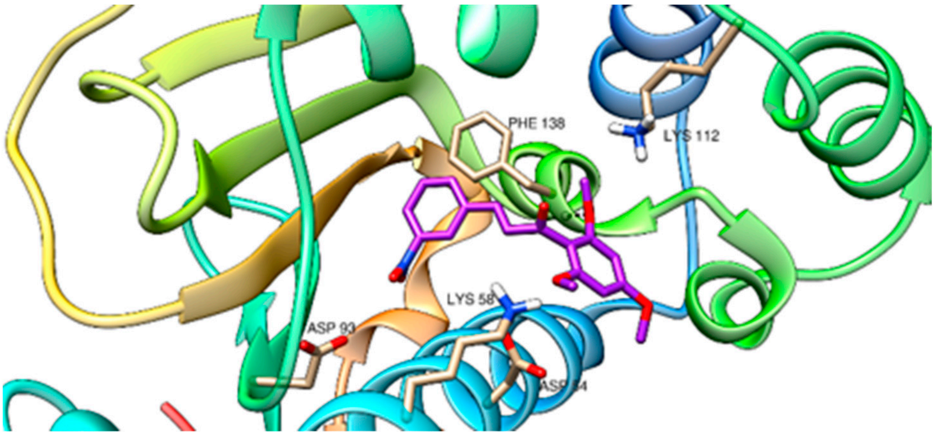

B

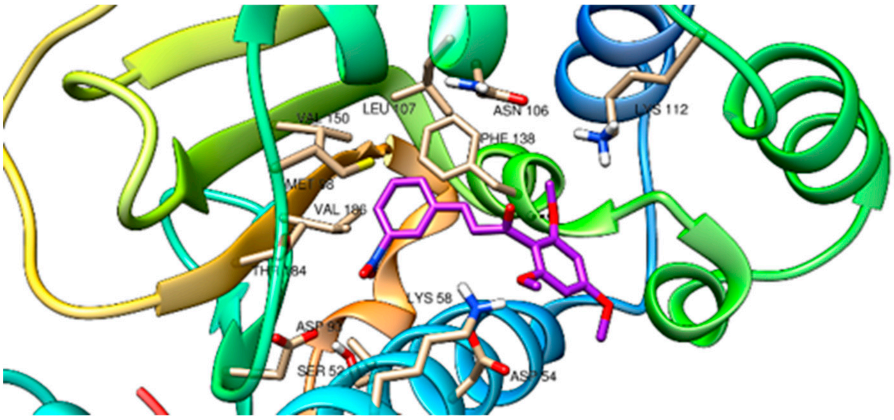

C

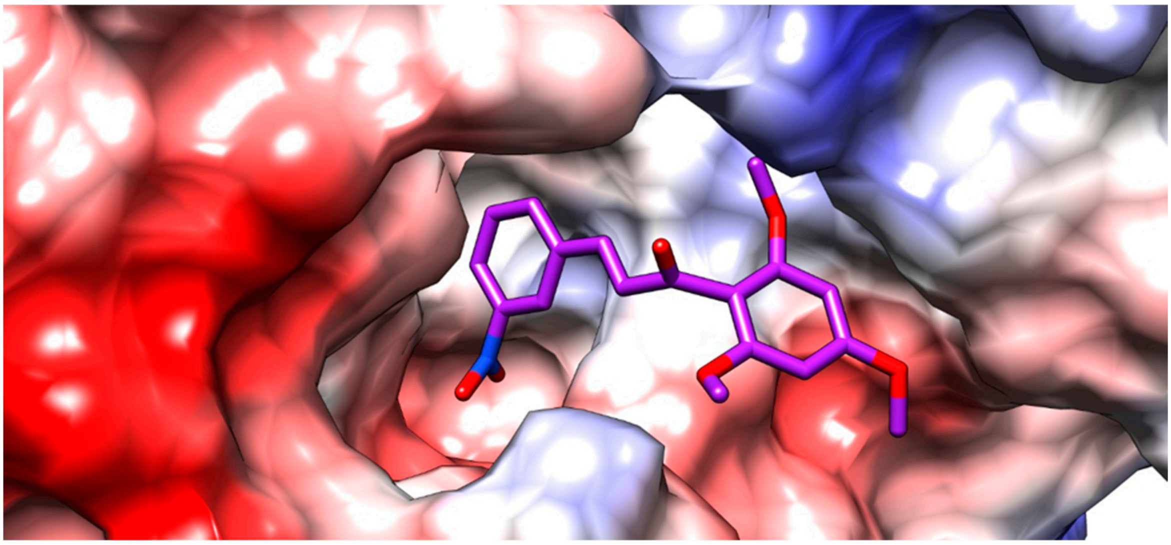

Figure S2. Molecular binding of SU086 to HSP90.
(A) SU086, a nitro derivative of 246TMP-3SF5, when docked in HSP90, shows different binding mode compared to 246TMP-3SF5. It has an affinity score of -7.3 kcal/mol. **(B)** SU086 docked in HSP90 for reference to show the nitro group flipped from hydrophobic pocket to hydrophlic pocket. SU086 shows interaction with Phe138. **(C)** The surface view of SU086 shows phenyl–NO_2 group is facing towards Asp54 and Ser53, which gives polarity to the local area, giving stability to the molecule. In the case of 246TMP-3SF5, the –SF5 group was facing upwards, where all hydrophobic groups are residing like Met98,

Leu107, Val150 and Val186. Owing to the rigid conformation of chalcones, there is change in binding mode of SU086.

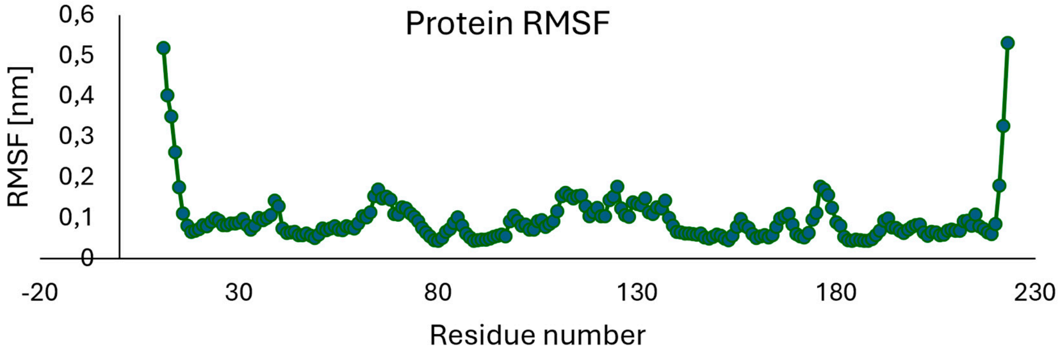

Figure S3. RMSF of protein backbone during MD simulation.

The root mean square fluctuations show high fluctuations in N-terminal and C-terminal loops of the protein, while α-helices and β-sheets do not show any major fluctuations, indicating that the secondary structure of the protein remains undistorted during simulation.
